# Streamline tractography of the fetal brain in utero with machine learning

**DOI:** 10.1162/imag_a_00537

**Published:** 2025-04-09

**Authors:** Weide Liu, Camilo Calixto, Simon K. Warfield, Davood Karimi

**Affiliations:** Boston Children’s Hospital and Harvard Medical School, Boston, MA, United States; Elmhurst Hospital Center and Icahn School of Medicine at Mount Sinai, New York, NY, United States

**Keywords:** tractography, machine learning, fetal brain, developing brain, diffusion MRI

## Abstract

Diffusion-weighted magnetic resonance imaging (dMRI) is the only non-invasive tool for studying white matter tracts and structural connectivity of the brain. These assessments rely heavily on tractography techniques, which reconstruct virtual streamlines representing white matter fibers. Much effort has been devoted to improving tractography methodology for adult brains, while tractography of the fetal brain has been largely neglected. Fetal tractography faces unique difficulties due to low dMRI signal quality, immature and rapidly developing brain structures, and paucity of reference data. To address these challenges, this work presents a machine learning model, based on a deep neural network, for fetal tractography. The model input consists of five different sources of information: (1) Voxel-wise fiber orientation, inferred from a diffusion tensor fit to the dMRI signal; (2) Directions of recent propagation steps; (3) Global spatial information, encoded as normalized distances to keypoints in the brain cortex; (4) Tissue segmentation information; and (5) Prior information about the expected local fiber orientations supplied with an atlas. In order to mitigate the local tensor estimation error, a large spatial context around the current point in the diffusion tensor image is encoded using convolutional and attention neural network modules. Moreover, the diffusion tensor information at a hypothetical next point is included in the model input. Filtering rules based on anatomically constrained tractography are applied to prune implausible streamlines. We trained the model on manually-refined whole-brain fetal tractograms and validated the trained model on an independent set of 11 test subjects with gestational ages between 23 and 36 weeks. Results show that our proposed method achieves superior performance across all evaluated tracts. Qualitative assessments on independent data from the Developing Human Connectome Project demonstrated the generalizability of our method. The new method can significantly advance the capabilities of dMRI for studying normal and abnormal brain development in utero.

## 1 Introduction

### 1.1 Background and motivation

Medical imaging techniques have played an increasingly prominent role in understanding the development of human brain in utero (Glenn, 2010;Malinger et al., 2004). Imaging modalities such as magnetic resonance imaging (MRI) are also becoming more popular in studying brain abnormalities prior to birth (De Asis-Cruz et al., 2022;Hosny & Elghawabi, 2010). As a consequence, fetal MRI has become an indispensable tool in medicine and neuroscience. As the fetal period represents a dynamic and vulnerable stage in brain development, the potential medical and scientific impacts of fetal brain imaging are enormous.

Among various medical imaging modalities, diffusion MRI (dMRI) has emerged as a unique method for studying the fetal brain (Hüppi & Dubois, 2006;Ouyang et al., 2019). It offers important insights into tissue microstructure and structural connectivity that no other imaging technique can provide. Research on adult brains has extensively documented that the microstructure of brain white matter tissue is a unique indicator of normal and abnormal brain development (Bodini & Ciccarelli, 2009;Salat, 2014). Moreover, structural connectivity of the brain can influence and be influenced by the progression of brain diseases (Collin & Van Den Heuvel, 2013;Fornito et al., 2015). These findings suggest that dMRI can play a crucial role in studying the fetal brain in-utero, where it undergoes its formative developments. Technical advancements in fetal dMRI have enabled tract-specific assessment of white matter (Deprez et al., 2019;Khan et al., 2018;Snoussi et al., 2024). There is also growing interest in quantitative assessment of the brain’s structural connectome in the fetal period (Jakab et al., 2015;Kasprian et al., 2013). Recent evidence suggests that the structural connectivity of the brain in utero can be altered by environmental factors and diseases. For example, prenatal exposure to maternal stress (Scheinost et al., 2017), congenital heart disease (CHD) (Schmithorst et al., 2018), and brain malformations (Jakab et al., 2015) may disrupt the normal development of the structural connectome in utero.

Despite these unique potentials, dMRI-based assessment of the fetal brain has progressed very slowly. This is in large part because of a lack of reliable computational methods to automate the image data analysis. For analyzing adult brain dMRI data, there exists a repertoire of reliable computational tools (Cai et al., 2021;Cieslak et al., 2021;Dubos et al., 2023;Garyfallidis et al., 2014;Joseph et al., 2021;Theaud et al., 2020;J.-D. Tournier et al., 2019). For the fetal brain, similar tools are almost entirely nonexistent. Existing computational methods that have been developed primarily for adult brains cannot be applied directly to analyze the fetal dMRI data. There are various factors that make fetal brain dMRI analysis different from adult brains. First, due to limited scan times to minimize maternal discomfort, low signal-to-noise ratio, and unpredictable motion, the data quality in fetal dMRI is typically much lower (Jakab et al., 2017;Marami et al., 2017). As a result, computation of parameters such as fiber orientation distribution is usually more noisy and less reliable. Moreover, because of incomplete myelination and relatively higher water content, fetal tissue microstructure is very different from that of the adult brain (Hüppi & Dubois, 2006;Pietsch et al., 2019). Additionally, the fetal brain undergoes rapid development within a short period. Tissue contrast changes dramatically during gestation and different white matter tracts emerge, develop, and myelinate at different points and different rates in this period (Dubois et al., 2014;Khan et al., 2019). Hence, the microstructure and configuration of fetal white matter tracts are quite different from those of a fully developed brain. Thus, successful fetal brain tractography requires dedicated computational methods that currently do not exist.

This gap in technology has seriously limited our ability to tap the potential of dMRI to study brain’s earliest and most critical developments. As an example, the only study to assess the impact of CHD on fetal brain white matter relied on manual tract delineation (Khan et al., 2018). Because of the time and expertise requirements, that study was limited to one single tract and only a handful of subjects at one single gestational age. Automatic computational methods specifically tailored to fetal brain data can drastically enhance our ability to analyze larger fetal cohorts across the gestational age. Such tools can also reduce the cost and time requirements of these studies, increase the accuracy, and improve the reproducibility compared with manual annotations.

The goal of this work, therefore, was to develop and validate a new method for tractography of the fetal brain. Given the recent success of machine learning for this application (Neher et al., 2017;Poulin et al., 2019), our proposed method is based on a deep learning model that is trained on manually refined tractography data. Compared with existing tractography methods, there are several technical aspects that make our method better suited for fetal brain tractography. First, our method is based on a machine-learning model that is trained on a set of streamlines computed from real fetal dMRI scans and verified by human experts. Second, to reduce the reliance on local fiber orientation at the current tractography propagation point, our method utilizes a large spatial context around the current point, which is encoded into multi-scale feature maps using convolutional and attention-based neural networks. Additionally, the input to our model includes a long history of prior propagation directions and information about the position of the current point within the brain, encoded as its distance to certain keypoints in the cortex. These inputs serve as useful anatomical information that can help the model compute more accurate streamlines in the presence of noisy local fiber orientation estimations. Moreover, our model leverages two fetal-specific information, that is, a fetal brain tissue segmentation map and a registered atlas of major fixel directions. Our model is trained to compute the next streamline propagation direction based on these inputs. Combined, these design features facilitate the computation of accurate streamlines throughout fetal gestation.

If successful, such a method can greatly improve the accuracy and reproducibility of quantitative fetal brain studies with dMRI. It can lead to more precise assessment of specific white matter tracts. Moreover, it can be used to reconstruct the structural connectome and to quantify the structural connectivity metrics in the fetal period. Accurate tractography may also aid in delineation of specific white matter tracts with automatic or semi-automatic methods (Garyfallidis et al., 2018;Suarez et al., 2012;Wassermann et al., 2016).

### 1.2 Related works

Bundle-specific and whole-brain tractography are widely used for delineating white matter tracts, for studying the white matter as a whole, and for reconstructing the structural connectome (C.-H. Yeh et al., 2021;F. Zhang et al., 2022). Early tractography algorithms relied on elementary models of the diffusion signal to compute the local fiber orientations and were primarily intended for qualitative or visual purposes (Basser et al., 2000). Over the past two decades, more advanced tractography algorithms have been developed (Li et al., 2014;Sepasian et al., 2012;Smith et al., 2012;F.-C. Yeh et al., 2019). A representative example of these advancements is the class of global tractography algorithms (Christiaens et al., 2015;Lemkaddem et al., 2014;Mangin et al., 2013), which treat tractography as a global inverse problem. Additional non-local information, such as anatomical context and history of streamline propagation, are often used by these advanced methods to improve the accuracy and reduce the false positive rates. There have been multiple efforts by the dMRI community to rigorously assess the capabilities and limitations of modern tractography algorithms (Girard et al., 2023;Maffei et al., 2022;Maier-Hein et al., 2017). Some of the persistent challenges in tractography include fiber crossings and bottlenecks, inherent limitation of the dMRI data for inferring the local white matter fiber orientations, and paucity of ground-truth data to develop and validate these methods (Rheault et al., 2020;F. Zhang et al., 2022).

Machine learning may hold the key to addressing some of the perennial issues in tractography. A primary advantage of machine-learning methods is their ability to integrate diverse sources of information, such as spatial information and anatomical priors. Classical non-machine-learning tractography techniques can also incorporate such auxiliary information. However, it would be highly challenging to design those methods such that they optimally leverage different inputs. For example, the utility of different inputs likely depends on the streamline being traced and the position along the streamline. Conventional tractography methods do not have the capacity to learn such complex relations. Modern machine-learning models such as deep neural networks, on the other hand, can learn these relations from training data. They can optimize their prediction objective with respect to different inputs much more effectively in a unified framework. Consequently, recent years have witnessed a growing interest in using machine learning for tractography. Reviews of these works can be found inKarimi and Warfield (2024)andPoulin et al. (2019).

Compared with adult brain studies, in utero fetal brain tractography has been much less utilized. This has been mainly because of the challenges of fetal dMRI acquisition and analysis. Compared with adult dMRI data, fetal brain dMRI measurements have overall inferior quality due to such factors as lower signal-to-noise ratio and motion effects. Moreover, different white matter tracts emerge and develop rapidly over a short span of time during the second and third trimesters. Due to these challenges, most prior works on fetal tractography have focused on one or a few selected tract bundles and, yet, have reported low reconstruction success rates and limited accuracy in reconstructing the tracts in their complete spatial extent (Kasprian et al., 2008;Kolasinski et al., 2013;Mitter, Prayer, et al., 2015;Takahashi et al., 2014;Zanin et al., 2011). Despite these limitations, prior works have also demonstrated that tractography has a great potential for studying normal and abnormal brain development in utero and for characterizing brain pathologies prior to birth (Huang et al., 2009;Jakab et al., 2015;Kasprian et al., 2008;Mitter, Jakab, et al., 2015;Mitter, Prayer, et al., 2015;Wilson et al., 2021;Xu et al., 2014;Zanin et al., 2011). Therefore, accurate and reproducible fetal tractography will not only serve quantitative assessment of structural connectivity, but it can also advance the field of fetal brain imaging in other important directions.

### 1.3 Summary of the contributions of this work

This work presents the development and validation of a machine-learning approach to streamline tractography of the fetal brain. Our proposed method follows design principles that make it well suited to the challenging nature of fetal dMRI data.

We compute the local orientation of white matter fibers using a diffusion tensor fit to the dMRI signal. This enables the method to be used with typical dMRI scans that may not be suitable for determining complex fiber configurations. We use a neural network model, consisting of convolution and attention modules, to encode a large spatial context around the current streamline tracing point in the diffusion tensor image. In addition to the current streamline tractography point, we will also include the diffusion tensor information at a hypothetical next point. We also encode the information about the position of the current streamline point in the brain mask in terms of the distance to selected keypoints in the brain cortex. Moreover, similar to several existing methods, our model input includes the directions of recent streamline propagation steps. The tissue segmentation map is also encoded and fed to the model. Finally, we precisely align a spatiotemporal atlas of major fixels to the subject’s brain and use that as additional input to the model. Our model synergistically combines this information to predict the next propagation direction.

We train the model on a set of whole-brain tractograms that have been manually edited and verified by human experts. Subsequently, we test our proposed method on a set of independent fetal brain scans. Our quantitative evaluations and visual assessments by an expert show that the proposed method achieves better results than a standard method and a machine learning technique. A comparison of our results with prior studies that have attempted tractography of the fetal brain shows that our results are vastly superior in terms of the range of white matter tracts that can be reconstructed and in terms of tract coverage. Therefore, our method represents a significant stride in improving the capabilities of dMRI for studying the brain in utero. The code will be available athttps://github.com/liuweide01/MLFT.

## 2 Methods

### 2.1 Image data acquisition and processing

This study used retrospective MRI data collected from fetuses scanned with 3T Siemens scanners at Boston Children’s Hospital. The study was approved by the institutional review board, and written informed consent was obtained from all participants. T2-weighted and diffusion MRI scans were collected from each fetus. The dMRI data for each fetus consisted of 2–8 acquisitions, each including one or two b = 0 images and 12–24 images with b = 500 s/mm^2^. Acquisition parameters were: repetition time (TR): 3,000–4,000 ms, echo time (TE): 60 ms, in-plane resolution: 2 mm, and slice thickness: 2–4 mm. Average scan time for T2-weighted and dMRI scans, each, was 10 minutes. The raw data were processed with a fetal-specific preprocessing framework (Marami et al., 2017) to correct for fetal/maternal motion and align all dMRI data into a standard space. As the standard reference space, we used a spatiotemporal atlas of the fetal brain. This atlas covers the gestational ages between 21 and 37 weeks and is publicly available (Gholipour et al., 2017). The fetal dMRI data processing pipeline, which has been extensively validated, has been described inKhan et al. (2019)andMarami et al. (2017). It performs slice-wise motion correction of dMRI data and registers the data to the age-matched atlas template. The pipeline combines all 2–8 acquisitions into one set of dMRI volumes, which are then used to perform the desired computations for the fetus. It can reconstruct dMRI volume with any desired resolution. We chose an isotropic voxel size of 1.2 mm in this work.

We fitted a diffusion tensor model to the data in each voxel. We then converted the diffusion tensor in each voxel to the diffusion orientation distribution representation (Descoteaux, 2008) and expressed it in spherical harmonics of order 8. As a result, the fiber orientation information in each voxel was represented with a vector of length 45. The reason for conversion from the diffusion tensor to diffusion orientation distribution was to facilitate the interpolation during tractography and to avoid the interpolation errors that may arise when working with tensors (H. Zhang et al., 2006).

We used multi-atlas segmentation techniques to generate tissue segmentation and gray matter parcellation maps for each fetus. For tissue segmentation, we used a manually segmented fetal diffusion tensor atlas (Calixto et al., 2023). For each fetus, we registered the tensor atlas to the subject using a diffusion tensor-based registration method (H. Zhang et al., 2006). For gray matter parcellation, we used a T2-weighted atlas of the fetal brain (Gholipour et al., 2017). We registered the atlas image to the mean diffusivity (MD) image of the subject. In both cases, we registered the three atlases that were closest in gestational age to the subject. Subsequently, we applied a probabilistic label fusion method (Akhondi-Asl & Warfield, 2013) to compute the final tissue segmentation and gray matter parcellation maps for the fetal subject. The tissue segmentation process involves classifying brain tissues into white matter, cortical gray matter, subcortical gray matter, and cerebrospinal fluid (CSF). These segmentations are essential for initiating and constraining the tractography process, especially for identifying and reconstructing complex white matter tracts.

Afterward, an expert visually inspected the diffusion maps (MD and FA), tensor glyphs (to ensure the accuracy of the principal eigenvector’s direction), and tissue segmentation maps for each subject, removing any poor-quality data or incorrect segmentation maps. From the initial cohort of 94 fetal subjects, a total of 73 subjects were deemed to have acceptable data quality. These fetuses represented a balanced distribution of gestational age between 23 and 36 weeks, with fetuses at all gestational weeks in that range. Of those, 62 subjects were allocated to the training set and 11 subjects to the test set.

To generate reference streamlines for model training, we applied a method based on streamline propagation using the iFOD2 algorithm (J. D. Tournier et al., 2010). Tractography seeding was performed on the boundary between gray matter and white matter, as described in more detail below inSection 2.6. We used a step size of 0.6 mm (equal to half the voxel size) and an angle threshold of$30^{\circ }$, which was selected empirically based on expert opinion to provide the most complete tractograms. We initially computed 5,000,000 streamlines for each fetus. Using the tissue segmentation maps, rules of anatomically constrained tractography (Smith et al., 2012) were applied to the iFOD2-generated results to remove implausible streamlines. As a result of applying these rules,11.6%±3.3%of the streamlines from each fetus were retained for training (that is, approximately580,000±165,000streamlines per training fetus). This percentage did not significantly depend on the gestational age. For fetuses under 30 gestational weeks and above 30 gestational weeks, respectively, the average was12.3%and10.2%. The final tractograms were reviewed by an expert to ensure that they did not include any gross errors.

### 2.2 External data

We also applied our method on fetal data from the Developing Human Connectome Project (DHCP). Compared with our in-house dataset used to train the proposed method described above, the DHCP data have been collected with a different scanner (Philips Achieva 3T) and different acquisition protocol (Christiaens et al., 2019;Price et al., 2019). One of the differences is that the b-values used in the DHCP dataset include b = 400 s/mm^2^and b = 1,000 s/mm^2^, while the b-value in our dataset was b = 500 s/mm^2^.

### 2.3 Method design

**Fig. 1.**
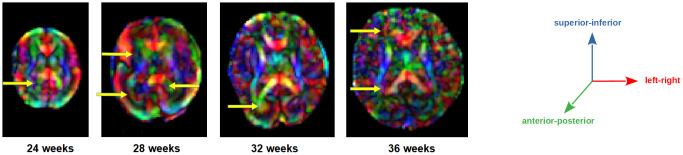
Color fractional anisotropy images for fetal brains scanned at 24, 28, 32, and 36 gestational weeks. The yellow arrows point to example regions with noisy or possibly erroneous values.

Figure 1shows color fractional anisotropy (color-FA) images and diffusion tensor glyph maps for four fetuses at 24, 28, 32, and 36 gestational weeks. These examples show that estimates of local fiber orientation can be very noisy and, on its own, not adequate for accurately guiding the streamline propagation.

To overcome this challenge, in this work we adopted several strategies. We briefly describe these strategies here and explain them in greater detail in the following subsections. First, we designed our method such that it leveraged the diffusion tensor information in a large spatial context around the current streamline propagation front. Second, we introduced additional local and non-local information to guide the streamline propagation. This information included recent propagation directions, local tissue segmentation map, and distance to standard keypoints in the brain cortex. Lastly, we used a spatiotemporal atlas of major fiber orientations, registered to the subject brain, to give the tractography algorithm the expected orientation of major fiber pathways. These strategies were meant to reduce the reliance on the estimate of local fiber orientation and enhance the robustness of the tractography results to the unavoidable errors in voxel-wise diffusion tensor fit.

**Fig. 2.**
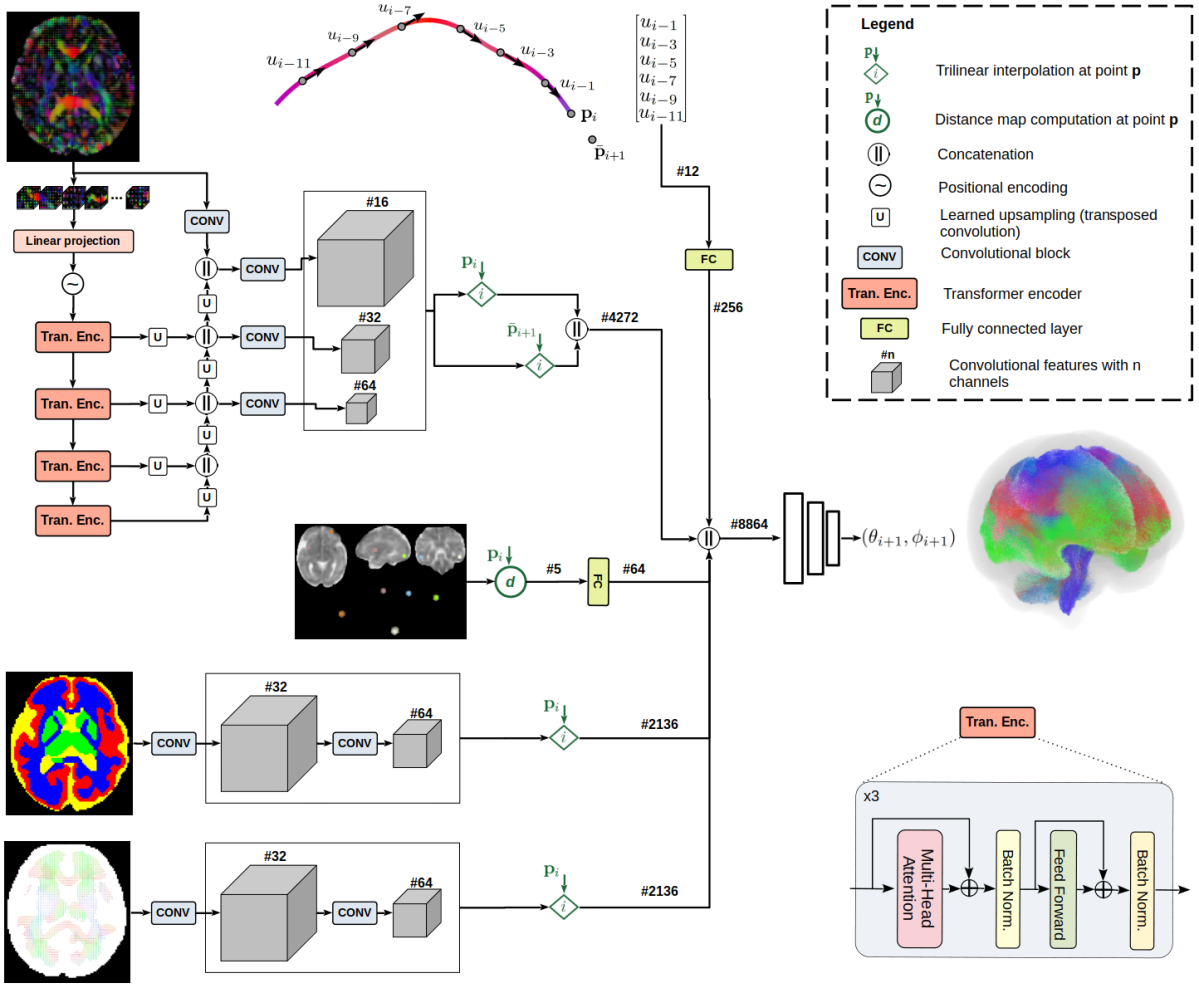
The proposed fetal tractography method. The method encodes the information in the 3D volume of diffusion orientation distribution using transformer and convolutional blocks, generating feature maps at three different scales. The spatial location of the current streamline propagation point is used to interpolate these feature maps. A similar procedure is followed to encode the information in the tissue segmentation map and the fixel atlas image registered to the subject, although using a light-weight fully-convolutional network. These are combined with prior streamline propagation directions and with “position vector” features that represent the global location of the current point in the brain. A set of fully-connected layers fuse these features to predict the next streamline propagation direction. The numbers specified after the # sign on each path indicate the dimensionality of the feature map on that path.

Figure 2shows the main components of the proposed method. Below, we first present the five sources of information that our method uses and explain how they are encoded. Subsequently, we describe how the method is trained and how it is applied to a test fetus.

#### 2.3.1 Fiber orientation information

The largest computational unit in our method consists of an encoder-decoder deep neural network based on convolutions and transformer blocks that encode the fiber information from the input diffusion tensor image. The input to this unit is the entire$128^{3}$-voxel image of diffusion orientation distribution computed from the diffusion tensor in each voxel. The image is divided into a set of$8^{3}$non-overlapping 3D patches. The patches are vectorized and projected into an embedding space. After positional encoding (Vaswani et al., 2017), the sequence of embedded patches is processed by a set of 4 transformer encoder blocks, each consisting of three transformer units with standard multi-head attention (Vaswani et al., 2017) and multilayer perceptrons. The design of the transformer encoder blocks largely follows the vision transformer (Dosovitskiy et al., 2020). Skip connections involving transposed convolution operations provide shortcut paths between the encoder and decoder sections of the network. The output of the decoder includes feature maps at three different scales:$F_{1}\left(H,W,D,C_{1}\right)$,$F_{2}\left(\frac{H}{2},\frac{W}{2},\frac{D}{2},C_{2}\right)$, and$F_{3}\left(\frac{H}{4},\frac{W}{4},\frac{D}{4},C_{3}\right)$, where$C_{1}$,$C_{2}$, and$C_{3}$denote the number of channels at each level. In this work, we set$C_{1}=64$,$C_{2}=128$, and$C_{3}=256$. Larger values for these hyperparameters would increase the model size and its representational power, but also increase the risk of overfitting. We chose these hyper-parameter values empirically to achieve a good trade-off between representational power of the model and risk of overfitting. The highest resolution feature maps,$F_{1}$, include features computed with a set of convolutional operations applied directly on the input diffusion orientation distribution image.

The feature maps thus computed provide a rich encoding of the diffusion orientation distribution image. Because of the deep and multi-scale design of the network, at each point in the image space the feature maps encode information about fiber orientation distribution from a large spatial extent around that point. For tractography, these feature maps need to be interpolated at the arbitrary location of the current streamline propagation point. We accomplish this via trilinear interpolation based on the voxels within a3×3×3grid around the current point. Additionally, we use the values of the features at the centers of voxels within a3×3×3-grid around the current tractography point. These values are concatenated to the interpolated values. To avoid a cluttered schematic, these last features are not shown inFigure 2.

Additionally, we extracted features computed from the diffusion orientation distribution image at a hypothetical next point. Specifically, let us denote the current point with${\text{p}}_{i}$and the direction of the last propagation with${\text{u}}_{i-1}$. We compute the hypothetical next point as${\bar{\text{p}}}_{i+1}={\text{p}}_{i}+\delta {\text{u}}_{i-1}$, whereδis the look-ahead step size that we set to 0.5 voxels in all experiments. In essence,${\bar{\text{p}}}_{i+1}$would be the next streamline point if the current propagation direction were to be the same as the last propagation direction, that is, if we keep propagating in the same direction. Similar ideas have been advocated for in prior works. For example,J. D. Tournier et al. (2010)use a similar mechanism to obtain a look-ahead view of how the fiber orientation distribution looks like in front of the streamline being traced. We interpolated the feature maps extracted from the diffusion orientation distribution image at${\bar{\text{p}}}_{i+1}$in a manner similar to that explained above for the current tractography point.

The features thus extracted from the diffusion orientation distribution image are concatenated and reshaped into a 1-D vector that is subsequently used, along with other inputs described below, by a multilayer perceptron (MLP) to compute the next propagation direction.

#### 2.3.2 Global location within the brain

Another piece of information that we feed to the model consists of an encoding of the position in the brain mask. This information can be useful because similar patterns of local fiber orientation may exist in different parts of the brain and correspond to different white matter tracts. Encoding the global position of the current tractography point should be helpful in resolving such ambiguities and improving the reconstruction of different white matter tracts that may locally have similar shapes. Since the fetal brain grows dramatically, the challenge is how to achieve a consistent encoding of the position across the gestational ages. To address this challenge, we relied on brain parcellation labels. As mentioned above, we used the same parcellation scheme based on an existing spatiotemporal fetal brain atlas that covered all gestational ages (Gholipour et al., 2017). We selected five specific parcellation regions from this atlas: (1) Left orbital part of the middle frontal gyrus, (2) Right insula, (3) Right inferior occipital gyrus, (4) Right middle temporal gyrus, and (5) Left inferior temporal gyrus.

We selected these regions to be non-coplanar and far apart from each other across the gestational ages, as shown inFigure 3, in order to provide an unambiguous encoding of all positions within the brain mask. The minimum number of non-coplanar reference points to give an unambiguous representation of all positions in 3D is four. We arbitrarily chose five points because each point will add only 1 to the dimension of the corresponding feature vector, described below. We do not claim that these ROIs are optimal in any sense, but they serve our described criteria.

**Fig. 3.**
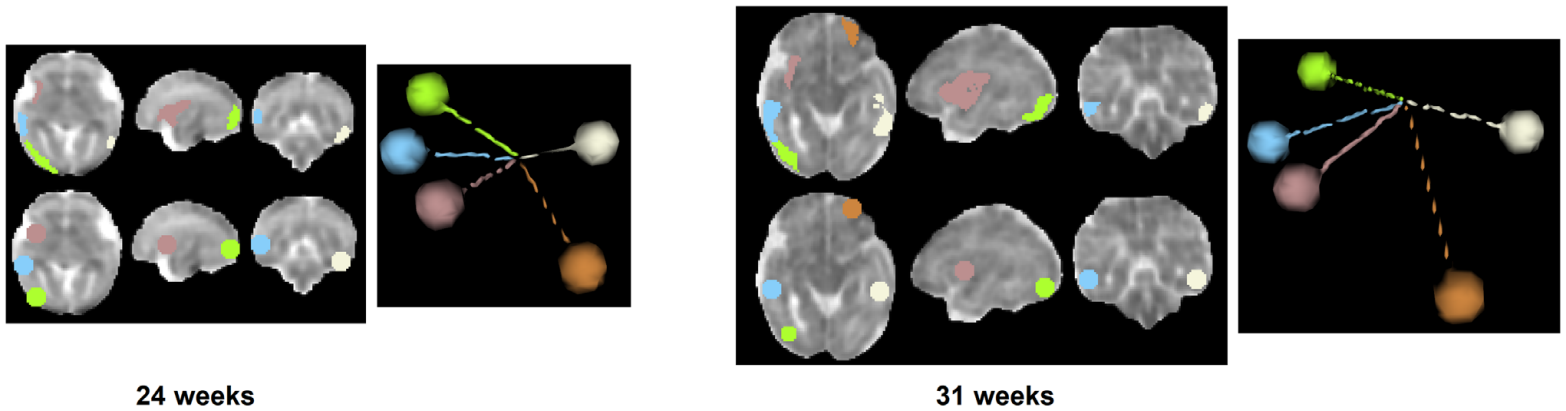
Our proposed scheme for encoding the position of the current tractography propagation point in the brain. We encode this information as the normalized distance with respect to the centers of mass of five non-coplanar cortical parcellation regions. This figure shows the axial, sagittal, and coronal views depicting the cortical parcellation regions that are visible in the shown slices, spheres denoting the centers of mass of those regions, and 3D views that show the lines connecting these centers to an arbitrary point within the brain mask. The fetuses shown in this figure are 24 and 31 weeks of gestational age.

Let us denote the center of mass of each of these parcellation regions with${\left\{{\text{c}}_{k}\right\}}_{k=1:5}$. We calculated the Euclidean distance from the current streamline position${\text{p}}_{i}$to${\text{c}}_{k}$as$r_{k}=∥{\text{p}}_{i}-{\text{c}}_{k}∥$and formed a normalized vector of these distances as the position encoding vector${\text{r}}_{i}$.

$${\text{r}}_{i}=\left[\frac{r_{1}}{\sum_{k=1}^{5}r_{k}},\frac{r_{2}}{\sum_{k=1}^{5}r_{k}},\ldots ,\frac{r_{5}}{\sum_{k=1}^{5}r_{k}}\right]$$ (1)

This vector${\text{r}}_{i}$is passed directly to the final MLP.

#### 2.3.3 Streamline propagation history

An intuitively useful piece of information to guide the tractography is the streamline propagation history. This information has been used in some classical tractography algorithms as well. For example,Malcolm et al. (2010)used an unscented Kalman filter to combine the streamline propagation history and the dMRI signal at the current streamline point to estimate the next propagation direction.Lazar et al. (2003), on the other hand, proposed a tensor deflection method that changed the propagation direction of an incoming streamline based on the shape of the diffusion tensor in the current voxel. Leveraging the propagation history is also a common fixture of most machine learning-based tractography techniques.Poulin et al. (2017)andJorgens et al. (2018)directly incorporated the normalized vector of prior propagation directions as input to their model. Similarly,Neher et al. (2017)used the dot product of the prior direction as a weight for computing the next propagation direction in order to encourage alignment with the previous orientation. We have found that encoding the immediate prior propagation directions alone would make the model training difficult. Since most streamlines are locally very smooth, the immediate prior directions are often very close to the next target direction, making it difficult for the model to learn the small changes in direction between successive propagation steps. Hence, in our proposed method we skip some of the immediate prior steps and, instead, include earlier directions. Specifically, the propagation history that is used as input to our model includes$\left[u_{i-1},u_{i-3},u_{i-5},u_{i-7},u_{i-9},u_{i-11}\right]$.

#### 2.3.4 Tissue segmentation map

Streamlines are expected to start from brain gray matter, go through the white matter, and end in other gray matter regions. Therefore, segmentation of the brain tissue is highly informative for accurate tractography. Tissue segmentation maps are commonly used to reject anatomically invalid streamlines, for example, streamlines that prematurely stop inside the white matter or streamlines that cross voxels with cerebrospinal fluid (CSF). However, we think the tissue segmentation can be more effectively used to improve the accuracy of computing the propagation direction, rather than only for rejecting anatomically implausible streamlines. To this end, we use the tissue segmentation information in a small neighborhood around the current tractography point as input to our model.

We used a multi-atlas approach based on an existing diffusion tensor atlas of the fetal brain (Calixto et al., 2023) to compute a tissue segmentation map for each fetal brain. Since its initial publication (Calixto et al., 2023), this atlas has been extended to cover the gestational ages between 23 and 36 weeks. We encode this information via a set of three learnable convolutional layers that compute three feature maps at different resolutions as shown inFigure 2. We interpolate these feature maps at the position of the current streamline tractography point and pass the computed feature vectors to the final MLP.

#### 2.3.5 Spatiotemporal fixel atlas

Due to the remarkable inter-subject similarity in brain structures, atlases are widely used to improve, constrain, and regularize the neuroimaging data analysis algorithms. They have also been used in a few prior studies on adult brain tractography. For example,Durantel et al. (2025)reconstructed a fixel atlas and used it as a prior to enhance the voxel-wise estimation of fiber orientation distribution (FOD). In each voxel, they computed an “enhanced FOD” using a weighted combination of the FOD computed from the dMRI signal and the fixel prior, where voxel-wise metrics such as generalized fractional anisotropy were used to compute a heuristic weight. The enhanced FOD was then fed to a standard (non-machine learning) tractography method. A similar approach was followed byRheault et al. (2019), where the atlas (prior) and subject FODs were simply multiplied in each voxel. However, by using the atlas value in the current voxel only, these methods fail to fully exploit the information in the atlas.

In our method, we first reconstructed a spatiotemporal atlas of major white matter orientations in each voxel (i.e., fixels). The atlas was computed by applying these operations on the training data. (1) We computed whole-brain tractograms for each fetus. (2) Diffeomorphic diffusion tensor-based registration was used to align the tractograms for all fetuses of the same age into a common space. (3) Clustering algorithms were used to detect the directions of major fixels in each voxel based on the streamline directions in that voxel. Details of the method are described inCalixto, Soldatelli, Li, et al. (2024). Example fixel maps are shown inFigure 4. We found that only 2% of the voxels contained more than two fixels. Therefore, in this work we used the directions of the first two fixels as additional input to our machine learning-based tractography model.

**Fig. 4.**
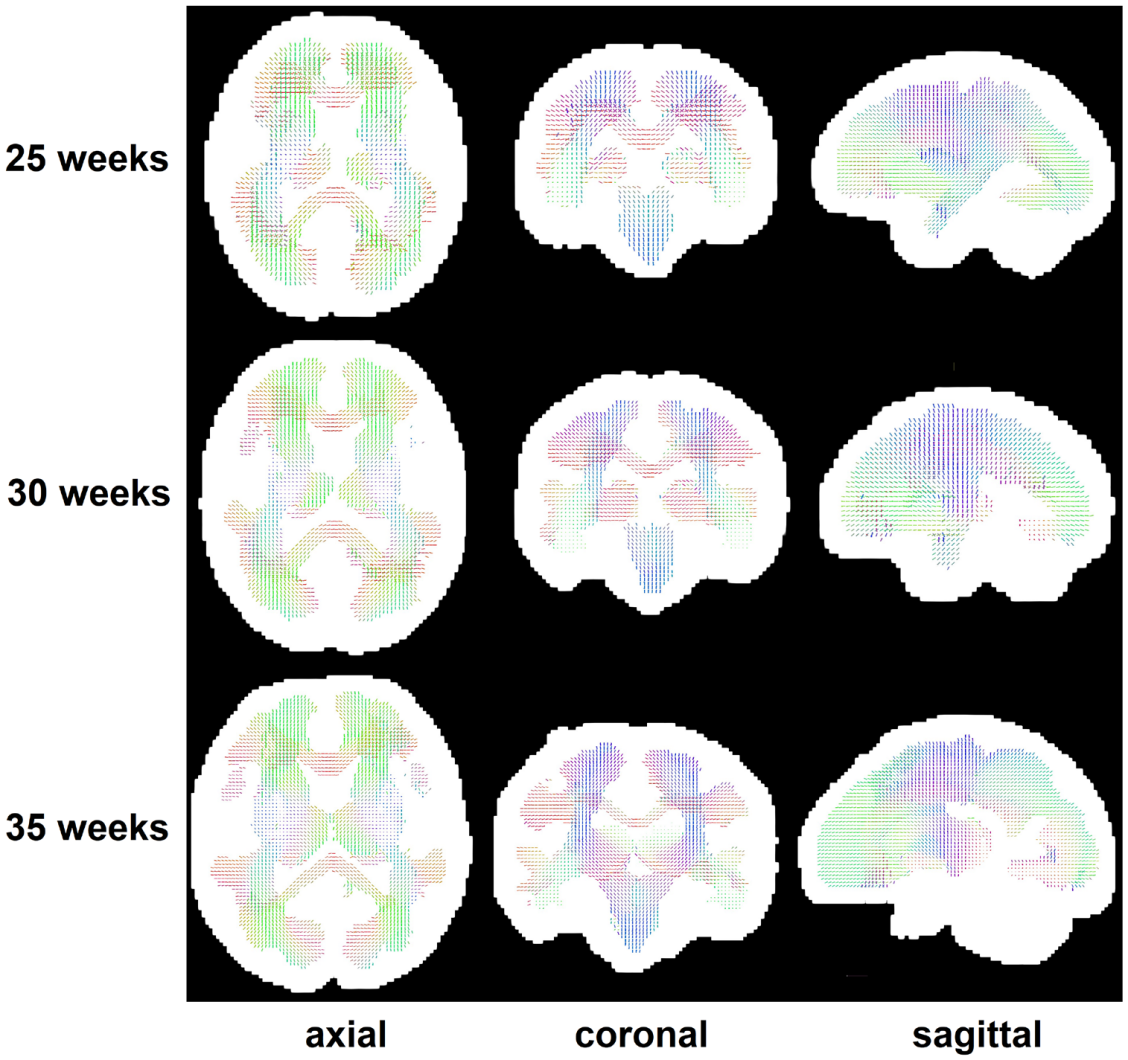
Axial, coronal, and sagittal views of the spatiotemporal fixel atlas for 25, 30, and 35 gestational weeks. In order to better view the atlases for lower gestational ages, all atlases have been displayed to the same size.

For each fetus, we register the age-matched fixel atlas using deformable diffusion tensor-based registration. As shown inFigure 2, unlike prior works that have used a simple voxel-wise weighting of fiber orientations based on the atlas, we include the atlas information along with other information in our machine-learning method. In other words, our proposed method computes the next streamline propagation direction by including the fixel atlas as one among many sources of information. We encode the fixel atlas information in a manner quite similar to the encoding of the tissue segmentation map. Specifically, we compute three sets of convolutional features at three different scales. We interpolate these feature maps at the location of the current tractography point and pass the computed feature maps directly to the final MLP for predicting the next propagation direction.

### 2.4 Training procedures

Our training dataset included a total of approximately 9 million streamlines from manually refined tractograms of 62 subjects. Although this is a notably large number, our preliminary experiments showed that a straightforward training on this dataset produced overly smooth streamlines at test time and did not allow the model to reconstruct the tracts with varying curvatures. A simple data augmentation approach to increase the diversity of the target propagation direction resolved this issue. To apply this augmentation, we assumed the streamline directionufollowed a von Mises-Fisher (vMF) distribution$u∼p_{\text{vMF}}\left(u;\mu ,\kappa \right)=C\left(\kappa \right)\text{ exp}(\kappa \mu^{⊤}\text{u}$). Here,μandκ, respectively, denote the mean direction and concentration parameter of the distribution andCis the normalizing coefficient. This is a standard model for representing distributions on the sphere and has been used in prior tractography works as well (Wegmayr & Buhmann, 2021). Minimizing the negative log likelihood of the observed direction gives rise to a simple loss function of the form$-\kappa \cdot \langle u,\hat{u}\rangle -\text{log}C$, where〈.,.〉denotes the inner product. This formulation encourages the closeness of the predicted orientation with the target orientation while allowing for learned weighting of data with high uncertainty via modulating the loss term withκ. In other words, for data with higher uncertainty (lowerκ) the model is allowed to make larger prediction errors by paying a higher “uncertainty penalty”. Note that, due to the negative sign, the optimization goal is to*increase*$\kappa \cdot \langle u,\hat{u}\rangle$, which can be accomplished by increasingκand/or reducing the angle betweenuand$\hat{u}$. In the second loss term,Cis a function ofκand penalizes high uncertainties. This prevents degenerate training where the model computes high uncertainty for all data samples to arbitrarily reduce the first loss term.

Whileκmay also be predicted by the model via including it in the loss function as explained above, in this work we use a heuristic approach to setting its value. Specifically, we setκto be proportional to the square of fractional anisotropy:$\kappa =\alpha {\text{FA}}^{2}$. We empirically setαto 1,600. Most white matter voxels in our data had an FA value in the range [0.05, 0.25], resulting inκ∈[4,100]. For voxels with FA>0.20, which mostly represent voxels that are on major white matter tracts with little partial volume effect,κ>64. With the concentration factorκ=64, the 90% confidence interval around the mean direction in the vMF distribution is a cone of approximately$12^{\circ }$. For a voxel with FA = 0.10, which corresponds to smaller or less developed tracts or borders of major tracts with substantial partial volume effect,κ=0.16and the cone of 90% confidence interval around the mean direction has an angle of approximately$24^{\circ }$. Regions with higher FA generally represent the location of the major tracts with a single dominant fiber orientation, where the streamline propagation direction is less ambiguous. Regions with lower FA, on the other hand, generally represent voxels with low dMRI signal anisotropy due to significant partial volume effects or crossing fibers. Streamline propagation direction in these areas is less certain, which justifies increasing the value ofκ.

As shown inFigure 2, our model predicts the direction of the next streamline propagation step in terms of spherical coordinatesθandϕ, which can be converted to a unit vector that we denote as$u_{\text{pred}}$. Having fixedκbased on FA, we use the cosine similarity between the predicted and target propagation directions as the loss function. Specifically, our training loss function is$-<\;u_{\text{pred}},u_{\text{aug}}>$, where$u_{\text{aug}}$is the unit vector representing the direction of the training streamline after augmentation based on the vMF model as described above.

### 2.5 Implementation details

The proposed method was implemented using PyTorch and trained on an NVIDIA A6000 GPU. We used a large batch size of 16,000 to improve the training stability. Each training sample corresponds to one specific point on one specific streamline. The dimensions of some of the main feature maps / feature vectors for a training sample are shown inFigure 2. From the viewpoint of the final MLP, for example, each training sample consists of an input feature vector of size 8,864, computed from the various sources, and an output of size two ([θ,ϕ]), representing the next streamline propagation direction. Nonetheless, note that the final MLP and the other network modules (i.e., convolutions, transformer encoders) are all trained jointly. During training, we randomly select 16,000 data points on streamlines from one of the training fetuses and use the input and output corresponding to those points to train the model. For the next training iteration, 16,000 points from another random selection of streamlines, from another fetus, are used. We used a Stochastic Gradient Descent optimizer with an initial learning rate of$10^{-3}$. Training was stopped when the training loss plateaued or began to fluctuate. In our experiments in this work, this usually happened after approximately 100 training epochs. Training the model with all training subjects requires approximately 2 days. Computing a whole-brain tractogram for a test subject takes approximately 30 minutes.

### 2.6 Application of the trained model on a test scan

Once the model training is complete, it can be applied to compute the whole-brain tractogram on a test subject. We use the tissue segmentation map for both streamline seeding and for rejecting implausible streamlines. Valid streamlines should begin and end at the boundary between gray matter and white matter. We adopt a surface-based seeding approach by launching the streamlines from voxels on the boundary between gray matter and white matter, as shown inFigure 5. Given the tissue segmentation map, we consider every voxel in the cortical gray matter that has at least one neighboring white matter voxel. We consider the vector between the centers of the gray matter voxel and that of the neighboring white matter voxel as the initial streamline propagation direction and launch a streamline in that direction. In order to increase the number and diversity of streamlines, we augment the position and direction of the streamline launch. Based on our voxel size of 1.2 mm, we jitter the position of the seed point in the x, y, and z directions, uniformly, by [-0.6 mm, 0.6 mm]. We consider the initial direction in the spherical coordinate system with polar angleθand azimuthal angleϕ, and add random values uniformly sampled from[−30,30]degrees independently toθandϕ. Overall, the number of streamline seeds for each gray matter voxel depends on the number of its neighboring white matter voxels. Specifically, for each gray matter voxel, we launch five streamlines toward each of its white matter voxel neighbors, each initialized differently based on the random perturbations described above.

**Fig. 5.**
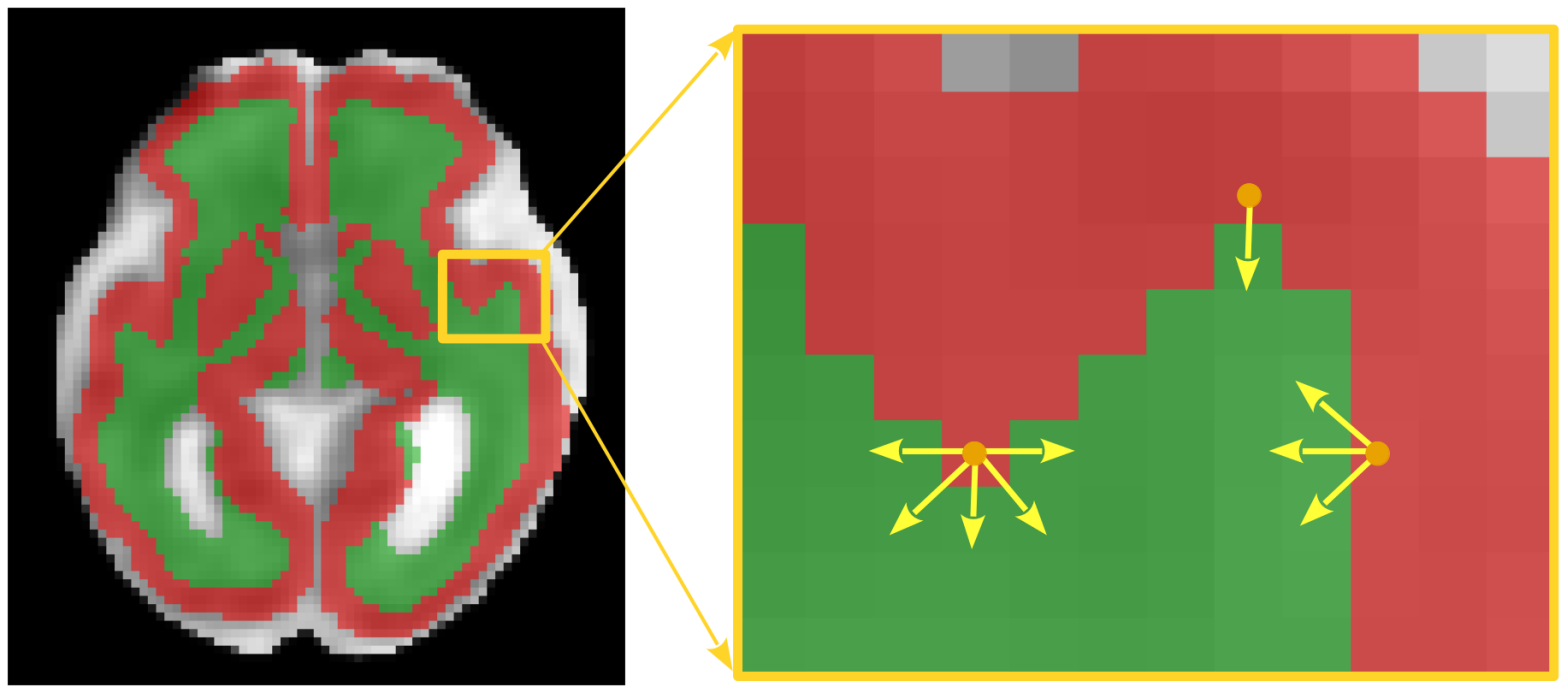
We launch a streamline from the center of each gray matter voxel that has at least one white matter voxel neighbor. The direction of the first step is selected to be the direction of the line connecting the center of the gray matter voxel to the center of the neighboring white matter voxel. In this figure, the red voxels are gray matter and the green voxels are white matter. We have selected three arbitrary gray matter voxels and have shown the direction of the first step for the streamlines launched from those seed points with yellow arrows. To enhance the tractogram diversity, random jittering is applied to the location of the seed point and the direction of the first step as explained in the text. These augmentations are not portrayed in this figure.

In order to enhance reconstruction of different tracts with varying shapes, we adopted a probabilistic tractography approach. Similar to the model training stage, we used a vMF distribution with the concentration factor modulated based on the local FA ($\kappa =\alpha {\text{FA}}^{2}$). Given the model’s prediction of the next propagation directionμ, we sample a random direction$\mu^{\prime }∼p_{\text{vMF}}\left(\mu ,\kappa \right)$and propagate the streamline along$\mu^{\prime }$by one step size. We used a step size of 0.6 mm, equal to half the voxel size for our data, in all experiments. We found that no single value ofαresulted in optimal reconstruction results for all tracts. While the settingα=1,600was quite adequate for reconstructing tracts with large maximum local curvature such as some sections of the corpus callosum, it was not the best setting for some other tracts with lower curvature such as the corticospinal tract. Therefore, on a test scan, we apply the method with three different values ofα∈[1600,3200,6400], to improve the reconstruction of various tracts. The final whole-brain tractogram for a test fetus is formed by simply merging the streamlines obtained with these three settings.

After propagating the streamline along$\mu^{\prime }$by one step size, the model is applied again to compute the next propagation direction. This process is continued as long as the next streamline point is inside the white matter segmentation mask. The propagation is terminated once the predicted next point is not inside the white matter. If the next point is on a cortical or sub-cortical gray matter voxel, the computed streamline is considered correct and it is added to the tractogram. If the next point is on a CSF voxel or outside the intracranial mask, the streamline is considered as anatomically implausible and rejected. Additionally, streamlines that exceed a length of 130 mm are discarded, as they exceed the expected maximum streamline length for the fetal brain.

### 2.7 Experiments and evaluation criteria

Assessment and comparison of tractography algorithms is notoriously difficult. In this work, we used both quantitative metrics and qualitative assessment by a human expert to evaluate the proposed method and compare it with existing techniques. In order to reduce the subjectivity of the assessments, we used an automatic tool, white matter query language (WMQL,Wassermann et al., 2016), to extract a set of anatomically meaningful white matter tracts from the computed whole-brain tractograms. This method uses standard definitions of the tracts and a parcellation map of the cortical and subcortical gray matter and white matter to automatically extract specific tracts. To generate the brain parcellation maps required by this tract extraction tool, we followed an atlas-based approach using a spatiotemporal atlas of fetal brain that covered the gestational ages between 21 and 37 weeks (Gholipour et al., 2017). We used the Symmetric Normalization (SyN) method from the Advanced Normalization Tools (ANTs) (Avants et al., 2008) to register the age-matched T2 atlas to the mean diffusivity image of each fetal brain. Subsequently, we used the computed registration transform to map the atlas parcellation to the subject fetal brain. We extracted nine tracts: Anterior Thalamic Radiation (ATR), Rostrum, Genu, and Splenium of the Corpus Callosum (${\text{CC}}_{1}$,${\text{CC}}_{2}$and${\text{CC}}_{7}$, respectively), Cortico-Spinal Tract (CST), Inferior Occipito-Frontal Fasciculus (IFO), Inferior Longitudinal Fasciculus (ILF), Optic Radiations (OR), and Uncinate Fasciculus (UF). Descriptions of these tracts can be found inCalixto, Soldatelli, Jaimes, et al. (2024).

We compared our proposed method with a conventional non-machine learning method, Fiber Assignment by Continuous Tracking (FACT) (Mori et al., 1999), and a recent machine-learning method based on recurrent neural networks (RNNs) (Cai, Lee, Newlin, Kim, et al., 2023). This RNN method was originally proposed for adult brain tractography and used a small spatial context of3×3×3voxels for the model input. Because our preliminary experiments had shown that a larger spatial context could be useful in fetal tractography, we also applied a variant of the RNN method where we increased the input patch size to7×7×7voxels. We refer to this method as “modified RNN”. For FACT, we used a cutoff value of 0.0001 as the stopping criterion and a maximum turning angle between successive steps of 50°, both selected empirically. For RNN and modified RNN we followed the implementation details suggested byCai, Lee, Newlin, Kim, et al. (2023). Similar to our method, for the compared methods we used a step size of 0.6 mm. Moreover, for compared methods we used the same seeding strategy as described above for our proposed method.

Our quantitative assessments were in terms of the Dice Similarity Coefficient, precision, and recall of the reconstructed tracts compared with the ground truth. In order to compute these metrics, we needed to convert the streamline bundle for an extracted tract to a binary mask. To this end, we first computed the streamline density map and then removed the voxels where the streamline density was below the 5^th^-percentile of the density values, which corresponded to spurious streamlines. To create the ground-truth binary tract masks for these evaluations, we generated masks from the iFOD2-computed streamlines, described inSection 2.1. An expert visually inspected each tract for every test fetus and marked the ones that were correctly and fully reconstructed. Only the tracts that passed the quality assurance were used in assessing our method and the compared methods in this work.

The qualitative evaluation by the human expert was carried out by scoring the tracts on a 1–5 scale using a bespoke scoring system. The expert visually inspected the tract in 3D and assigned a score from 1 (lowest score, indicating “failed reconstruction”) to 5 (highest score, indicating “excellent reconstruction”). The scoring system is described inTable 1. In these assessments, the tracts reconstructed by different methods were presented to the expert in a random order, and the expert was blind with respect to which method had reconstructed the tract being assessed.

**Table 1.** The scoring system used for qualitative assessment of the reconstructed tracts by a human expert in this study.

Score	Reconstruction quality	Description
1	Failed reconstruction	The method has failed to reconstruct any streamlines that could be considered as part of the expected tract.
2	Poor reconstruction	The method has reconstructed some streamlines that correspond to the tract of interest. However, the streamlines substantially deviate into incorrect paths and they may be very sparse. Overall, it is difficult to correctly identify the tract based on the streamlines.
3	Fair reconstruction	The reconstructed streamlines mostly follow the expected tract pathway, but there are notable deviations. The streamline paths and density, although overall plausible, suffer from small errors that lower its anatomical accuracy.
4	Good reconstruction	The computed streamlines closely match the tract’s anticipated anatomical path with high accuracy and sufficient density. Despite minimal errors, the streamlines are adequate to correctly define the tract’s outline.
5	Excellent reconstruction	The streamlines correctly and completely match the expected tract pathway. They precisely cover the entire spatial extent of the tract.

## 3 Results and Discussion

As explained inSection 2.6, from each gray matter voxel, we launched five streamlines in the direction of each of its white matter voxel neighbors. Depending on the volume of the fetal brain in our test data, approximately between 300,000 and 1,100,000 streamlines per brain were launched. We used the conditions of anatomically constrained tractography (Smith et al., 2012) to determine if a streamline was anatomically plausible. Of the streamlines computed with our method,17.3%±12%were anatomically plausible, while the rest violated those conditions and were thus rejected. For RNN, Modified-RNN, and FACT, the percentages of anatomically valid streamlines were, respectively,8.8%±7.0%,10%±8.9%, and3.9%±1.9%. The mean retention rate of 17% for our machine-learning method is significantly higher than the retention rate of approximately 4% for FACT. This suggests that the proposed method learns to compute more anatomically plausible streamlines, which not only improves the tractography accuracy but also reduces the computational time, the size of the tractogram, and the burden of subsequent tractogram post-processing operations. The percentage of valid streamlines for our method did not significantly depend on the gestational age. Specifically, the average percentage of anatomically valid streamlines for test fetuses under 30 gestational weeks was17.0%, while for fetuses above 30 gestational weeks it was17.9%.

### 3.1 Quantitative assessments

**Fig. 6.**
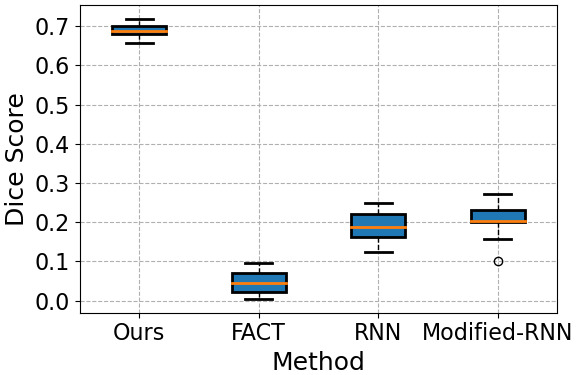

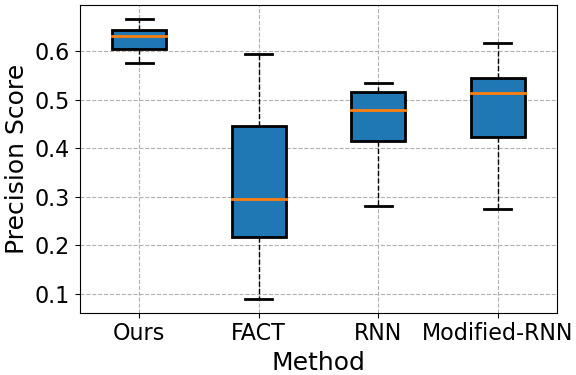

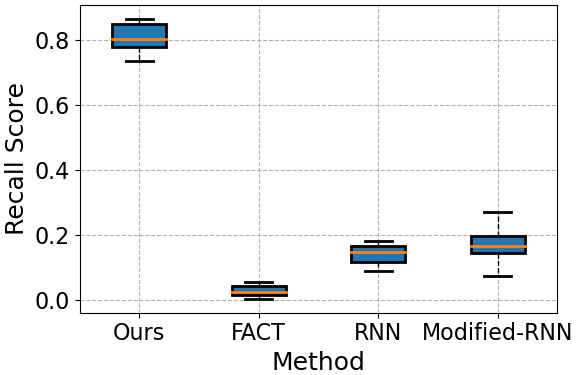
Summary of quantitative evaluation metrics for our proposed method and the compared techniques. The values shown in these plots have been pooled across 11 test subjects and 9 different white matter tracts.

Figure 6shows the results of quantitative evaluations in terms of DSC, precision, and recall. The plots in this figure show the summary of these metrics across all 11 subjects and all 9 white matter tracts. Across the 9 tracts and the 11 test fetuses, our method achieved a mean DSC of approximately 0.70, mean precision of above 0.60, and mean recall of above 0.80. Our method achieved substantially higher DSC, precision, and recall than FACT and the two RNN baselines. While the mean and standard deviation of the DSC for our method was0.711±0.027, for FACT, RNN, and Modified-RNN, respectively, it was0.048±0.028,0.190±0.039,and0.206±0.045.

**Fig. 7.**
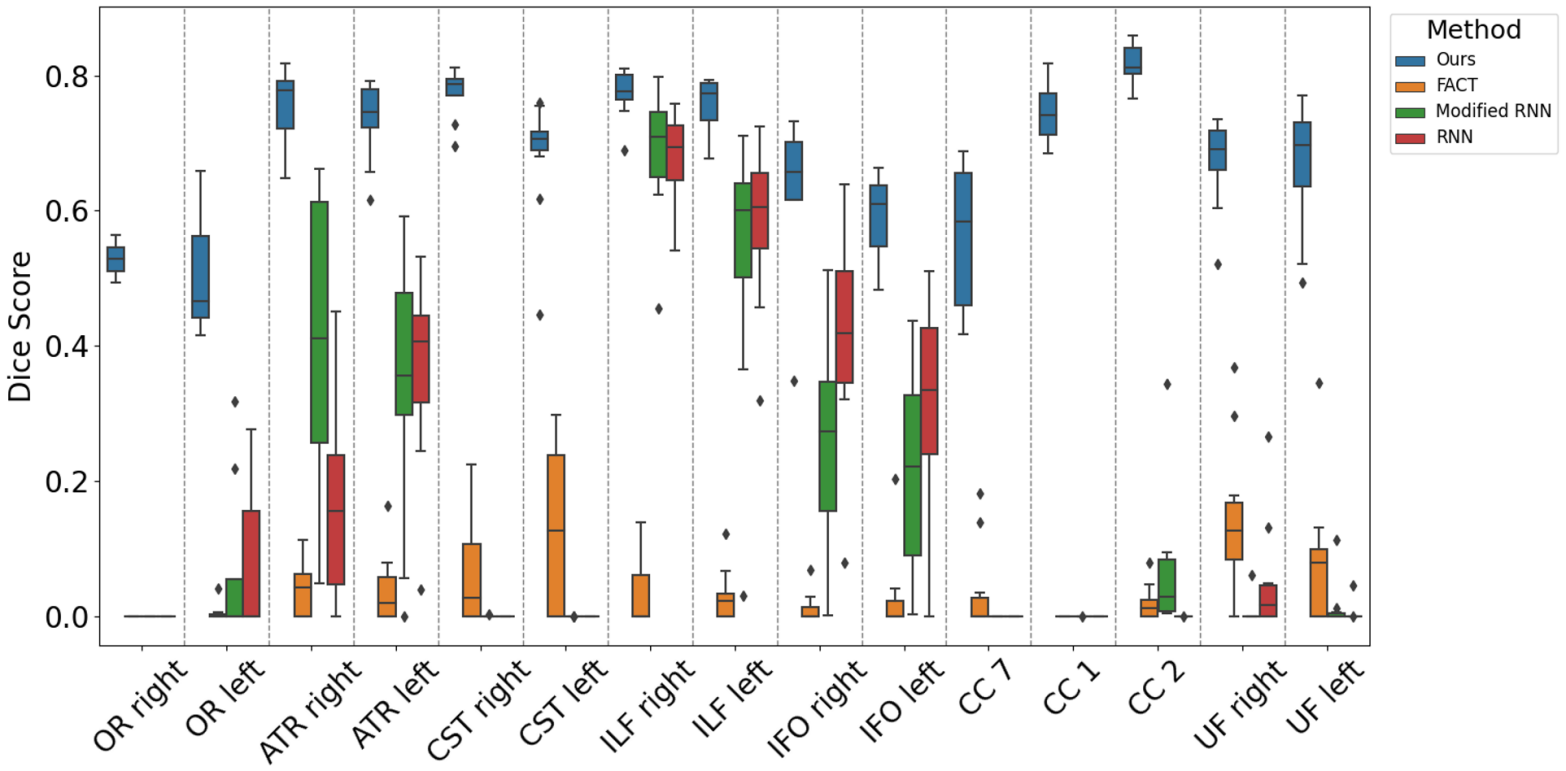
Performance of our proposed method and compared techniques in terms of the Dice score for individual white matter tracts.

Figure 7shows the reconstruction accuracy for our proposed method and the compared techniques in terms of DSC, separately for each of the tracts. For the bilateral tracts, we have shown the results separately for the left and right tracts. This figure shows that the proposed method can achieve a mean DSC of close to 0.50 or higher for all tracts, which is highly satisfactory given the challenging nature of fetal tractography. Although a direct comparison of our results with experiments on adult brains is not possible, our results compare very favorably with recent studies on adult brain tractography (Poulin et al., 2022). Nonetheless, the figure also shows significant variability in the reconstruction accuracy for different tracts. The proposed method has achieved mean DSC values of well above 0.70 for certain segments of the corpus callosum (${\text{CC}}_{1}$and${\text{CC}}_{2}$), CST, and some of the association tracts such as ATR and ILF. For other tracts such as OR and the splenium of the corpus callosum (${\text{CC}}_{7}$), on the other hand, the mean DSC is close to or below 0.60.

The variability in the reconstruction performance of the proposed method on different tracts may be attributed to at least three factors. First and foremost, being a machine-learning technique, the performance of the proposed method is ultimately restricted by the accuracy of the training data. Our experience shows that the ability of the proposed method to reconstruct a tract depends directly on the presence and completeness of that tract in the whole-brain tractograms that were used in model training. As an example, compared with${\text{CC}}_{1}$and${\text{CC}}_{2}$, the optic radiations (OR) was much less consistent in our training data. This has very likely contributed to the lower reconstruction accuracy for OR as shown inFigure 7. As another example, the observed lateralization of tractography results inFigure 7may be a manifestation of the impact of training data. We had noticed that the training tractograms used in this work were, in general, more accurate in the right hemisphere than in the left hemisphere. We speculated that this was likely due to the lower accuracy in the left brain hemisphere of the tissue segmentation maps that were used in computing the whole-brain tractograms for the training data. Interestingly, as shown inFigure 7, this has resulted in a consistently lower reconstruction accuracy for the left section of all the six bilateral tracts considered in this analysis. The impact of this factor may be ameliorated by improving the accuracy and reducing the bias of the training data.

The second factor that may have contributed to the differences in the reconstruction performance for different tracts may be the methodological choices and the algorithm settings. For example, as we have explained above, to generate the tractograms on test subjects we use a surface-based seeding approach, that is, we launch streamlines on the boundary between the white matter and gray matter. Although this is, in general, a suitable seeding strategy (St-Onge et al., 2018;F. Zhang et al., 2022), other seeding techniques may improve the reconstruction of certain tracts. Other settings in our method, such as the range ofαvalues, are likely to benefit the reconstruction of some of the tracts more than others. A simple remedy to this problem may be to expand the range of parameter settings used to compute the tractograms, but this will also inevitably increase the computation time and may also give rise to higher false positive rates.

Lastly, the inherent ambiguities in streamline tractography may impact the reconstruction accuracy of different tracts to different degrees. Some of the main sources of these ambiguities include the difficulty of resolving complex fiber orientations based on the dMRI signal in each imaging voxel, fiber crossings, and bottleneck regions (K. G. Schilling et al., 2019,^2022^). These factors are known to impact tractography in general and have been extensively studied for adult brains, but have received little systematic treatment for fetal brains (Calixto, Soldatelli, et al., 2024). Overall, tracts such as${\text{CC}}_{1}$that go through fewer fiber crossing and bottleneck regions should be easier to trace compared with tracts such as OR that encounter such ambiguities more often through their course. Compared with the other two factors mentioned above, this third factor relates to the fundamental limitations of dMRI-based fiber tracing that may not be completely surmountable. Nonetheless, it has been suggested that machine-learning methods such as the technique proposed in this work may represent one of the most promising approaches to addressing these limitations as well (K. G. Schilling et al., 2022).

### 3.2 Qualitative assessments

**Fig. 8.**
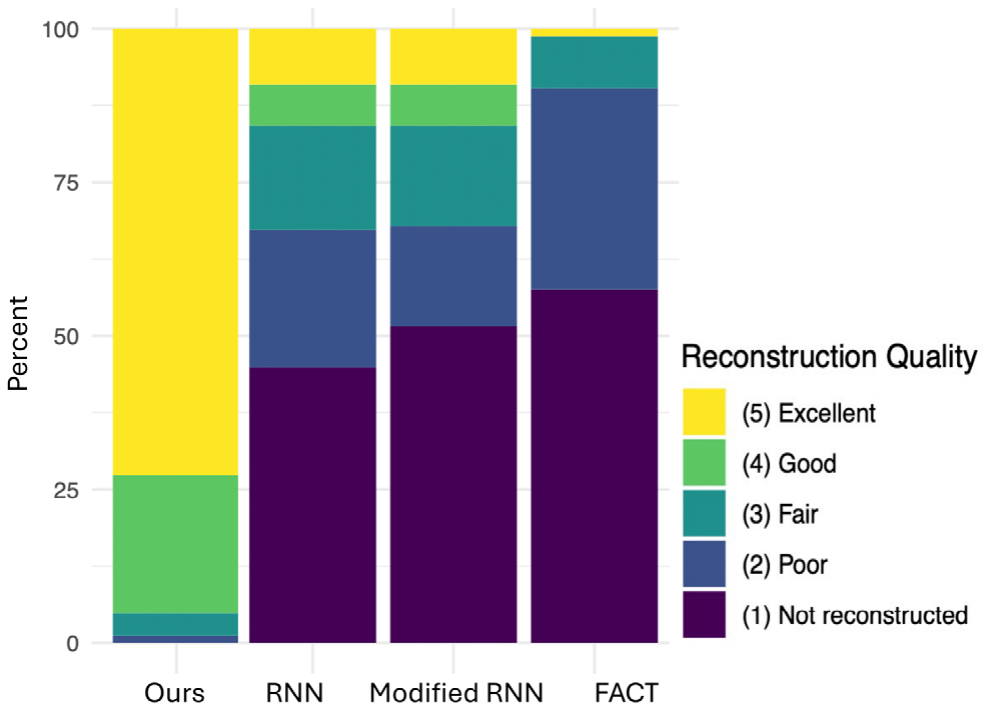
A summary of the quality scores assigned by an expert to the tracts reconstructed by the proposed method and the compared techniques. The results shown in this figure have been pooled across the 9 different tracts and the 11 test subjects. Descriptions of the scores are presented inTable 1.

Figure 8shows the results of expert ratings based on the scoring system presented inTable 1. This figure shows the summary of the scores across all 11 test subjects and all 9 white matter tracts considered in the assessments. On nearly 75% of the cases, our method received the highest score (Excellent reconstruction). This means that in the great majority of the cases, the tract reconstructed by our method precisely matches the expected anatomy with optimal streamline density and almost no spurious streamlines. In most of the remaining cases, the tracts reconstructed by our method received the second highest score on the scale (Good reconstruction), suggesting that the reconstructed tracts had only minimal deviations from the expected anatomy and the streamline density was adequate to demarcate the tract’s complete spatial extent.

The scores assigned to the tracts reconstructed by the other methods showed almost an opposite pattern. The great majority of the tracts were either not reconstructed at all or received a “Poor” score. This means that, in an expert’s opinion, the method either completely failed to reconstruct any valid streamlines or that the computed streamlines significantly deviated from the expected anatomy to the extent that it was not possible to identify the tract. This low performance was observed for the conventional method (FACT) as well as for the machine-learning baseline (RNN). These observations show the inability of existing methods to adequately address tractography of the fetal brain and clearly point to the difficulty of this application.

More detailed tract-specific scores are presented inFigure 9. They show that on all 9 tracts, the proposed method received an average score of above 4. This indicates that the method has accurately and faithfully reconstructed all tracts. RNN and Modified-RNN have accurately reconstructed the ILF and they have had moderate success in reconstructing ATR and IFO. However, they have performed very poorly in reconstructing the other six tracts. Similarly, FACT has failed to properly reconstruct any of the nine tracts. These results show that the proposed method can adequately reconstruct the full set of tracts considered in this study and that the existing techniques are clearly not well suited for this purpose.

**Fig. 9.**
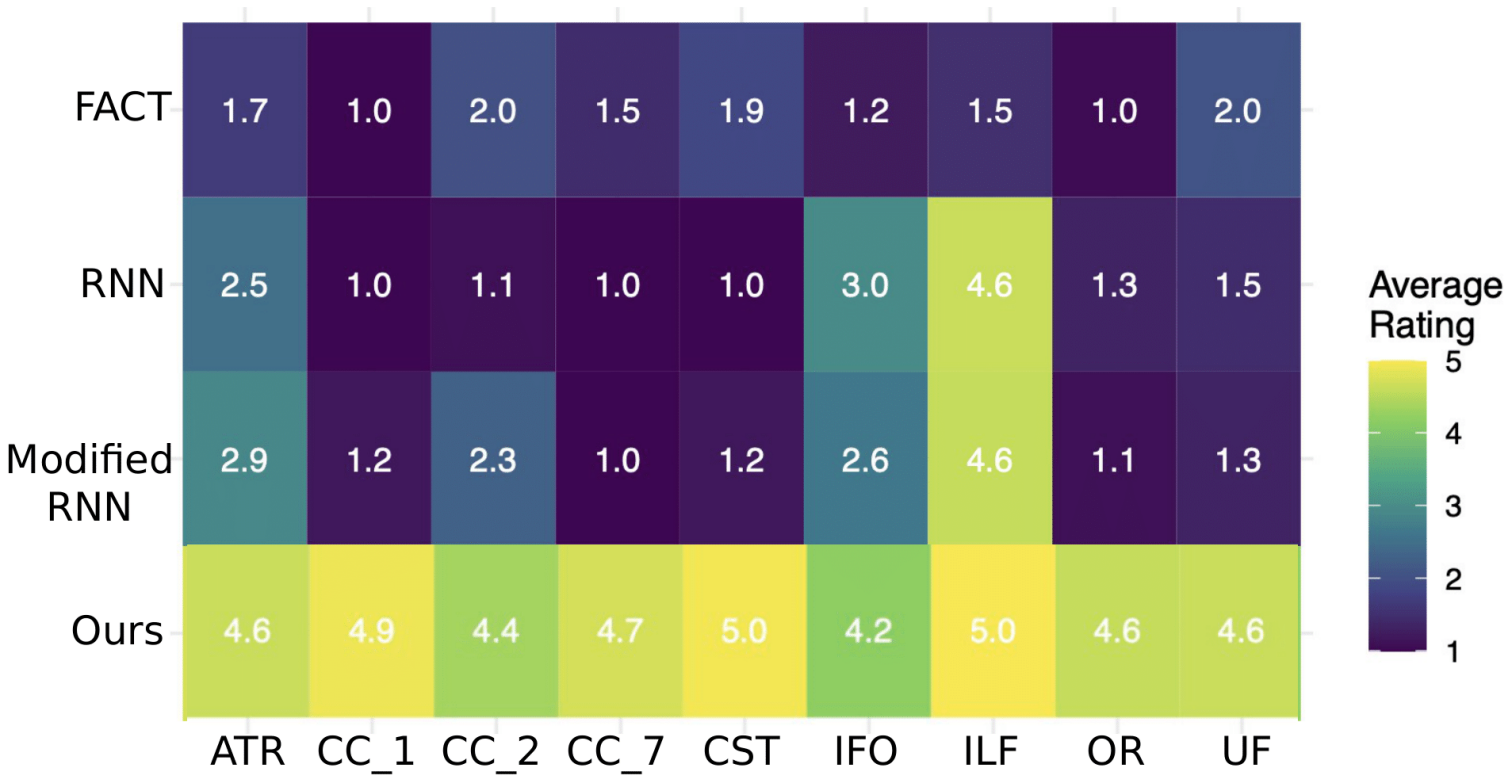
Detailed tract-wise reconstruction quality scores for the proposed method and the compared techniques. Descriptions of the scores are presented inTable 1.

A comparison of the results of this qualitative assessment with the quantitative assessment results presented in the above section can also be instructive. In particular, tracts such as OR and${\text{CC}}_{7}$that had received the lowest DSC scores in quantitative assessment have received high mean subjective scores of above 4.5 from the expert. This means that even though the overlap with the ground truth used for quantitative evaluation was not very high, in the expert’s judgment the streamlines reconstructed by our method completely covered the expected anatomical extent of the tract. This may point to the unavoidable errors and inherent limitations of the ground truth used for quantitative assessments. More importantly, it suggests that, despite the relatively low quantitative evaluation metrics for some of the tracts, the streamlines computed by our method may be sufficient to serve some of the most important applications such as tract-specific analysis and structural connectivity.

**Fig. 10.**
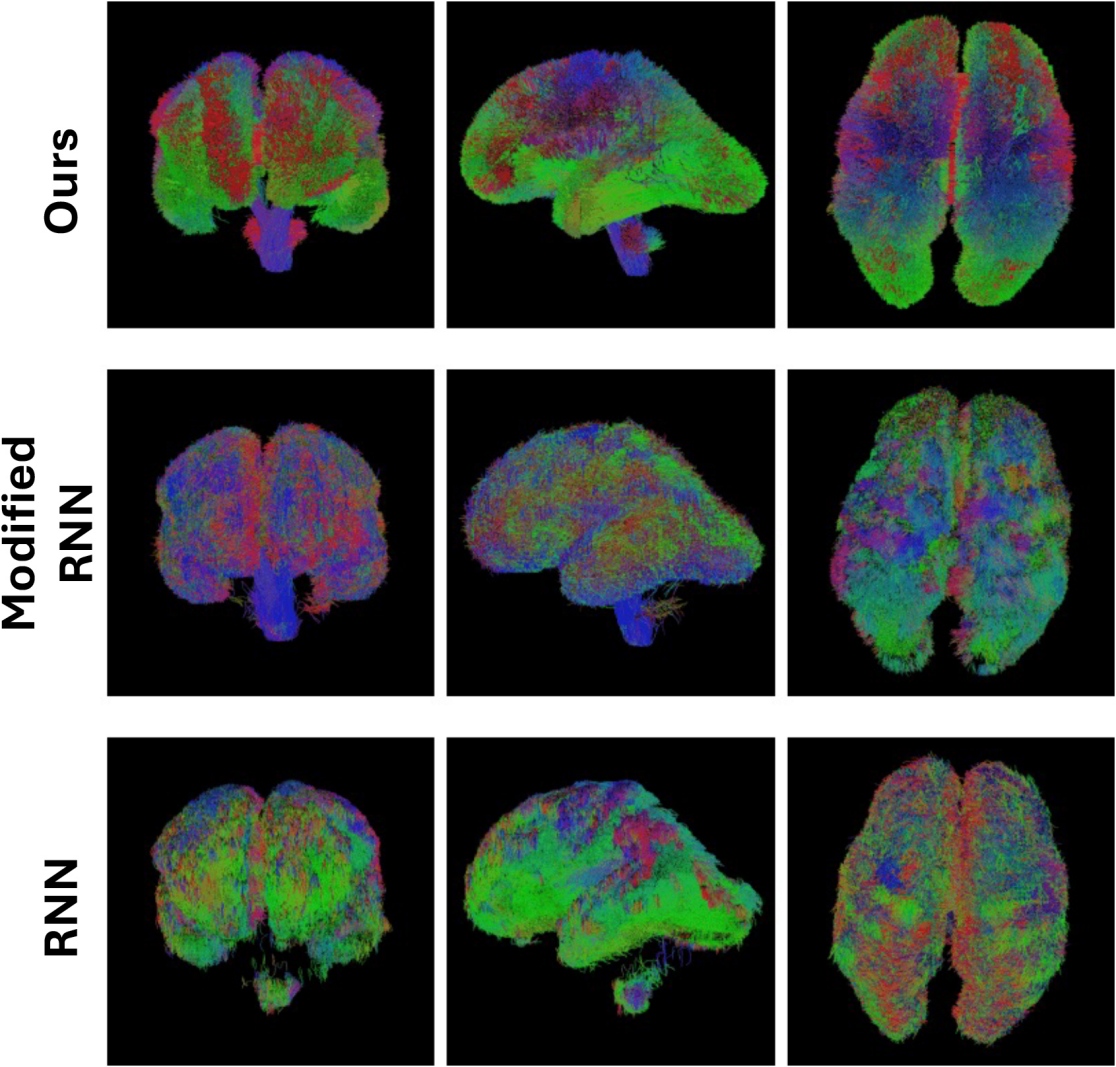
Visual comparison of whole-brain tractographies generated by our proposed method and other methods for a test fetus at 26 gestational weeks.

**Fig. 11.**
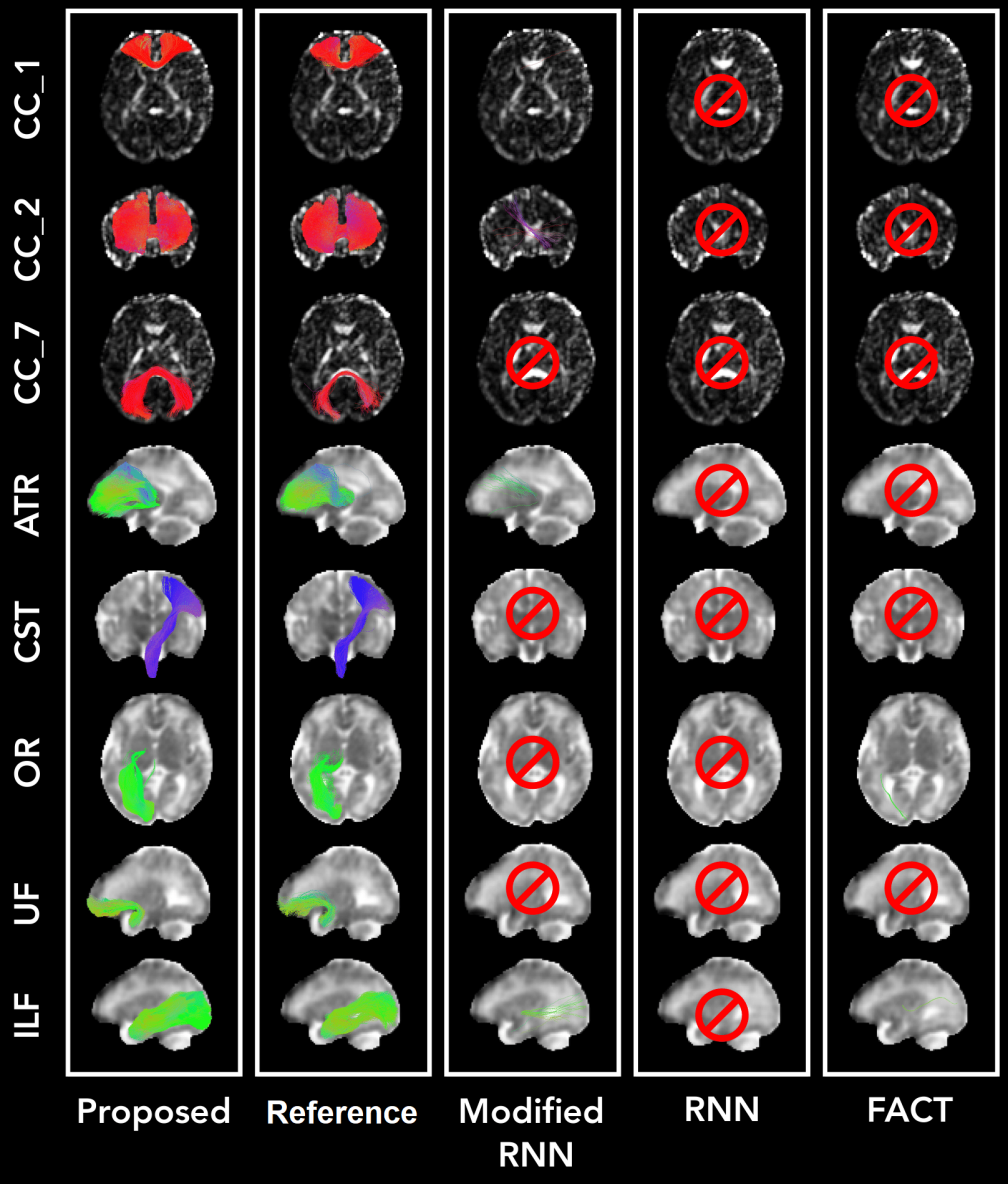
Comparison of tract reconstructions between our proposed technique and other methods on a 32-week fetus. In this example, our method reliably reconstructed all tracts, whereas other methods only managed to reconstruct a few tracts with low quality. Of note, for this specific test sample, RNN failed to reconstruct any of the tracts.

Visual comparisons of the results produced by our method and the compared techniques are presented inFigures 10and11.Figure 10portrays the whole-brain tractograms for a test fetus at 26 gestational weeks, whereasFigure 11depicts the individual tracts.Figure 10shows that the proposed method can reconstruct all regions of the tractogram. The results from the other two methods are not as good as our method. The RNN method, for example, has failed to reconstruct the brainstem. This may be due to RNN’s reliance on local fiber orientation information. At the level of cerebellum, the brainstem goes through regions with complex fiber configurations, which can be especially difficult to navigate due to the low quality of fetal MRI data. By leveraging non-local information and learning from large training datasets, our proposed method can avoid these difficulties.Figure 11shows that the proposed method can successfully reconstruct a range of association tracts (ATR, ILF, OR, IFO, and UF), commissural tracts (${\text{CC}}_{1}$,${\text{CC}}_{2}$, and${\text{CC}}_{7}$), and projection tracts (CST). The RNN and Modified-RNN methods have fair to moderate success in reconstructing some of the association tracts. However, they completely fail in reconstructing any valid streamlines for most tracts. FACT, on the other hand, has performed even more poorly than RNN and has failed to satisfactorily reconstruct any of the tracts.

Further visual comparisons are presented inFigure 12. This figure shows example results from our method compared with those computed by the reference method that has been used to generate the streamlines on the training data, as explained inSection 2.1. Similar to the results presented above, in this figure we have extracted the tracts using WMQL. These results show that our method can effectively learn to compute accurate streamlines and outperform the reference method that has been used to generate the training data on independent test samples. Although the reference method overall performs well, in some cases it completely fails to reconstruct the tract, while our method can reconstruct the same tract quite accurately.

**Fig. 12.**
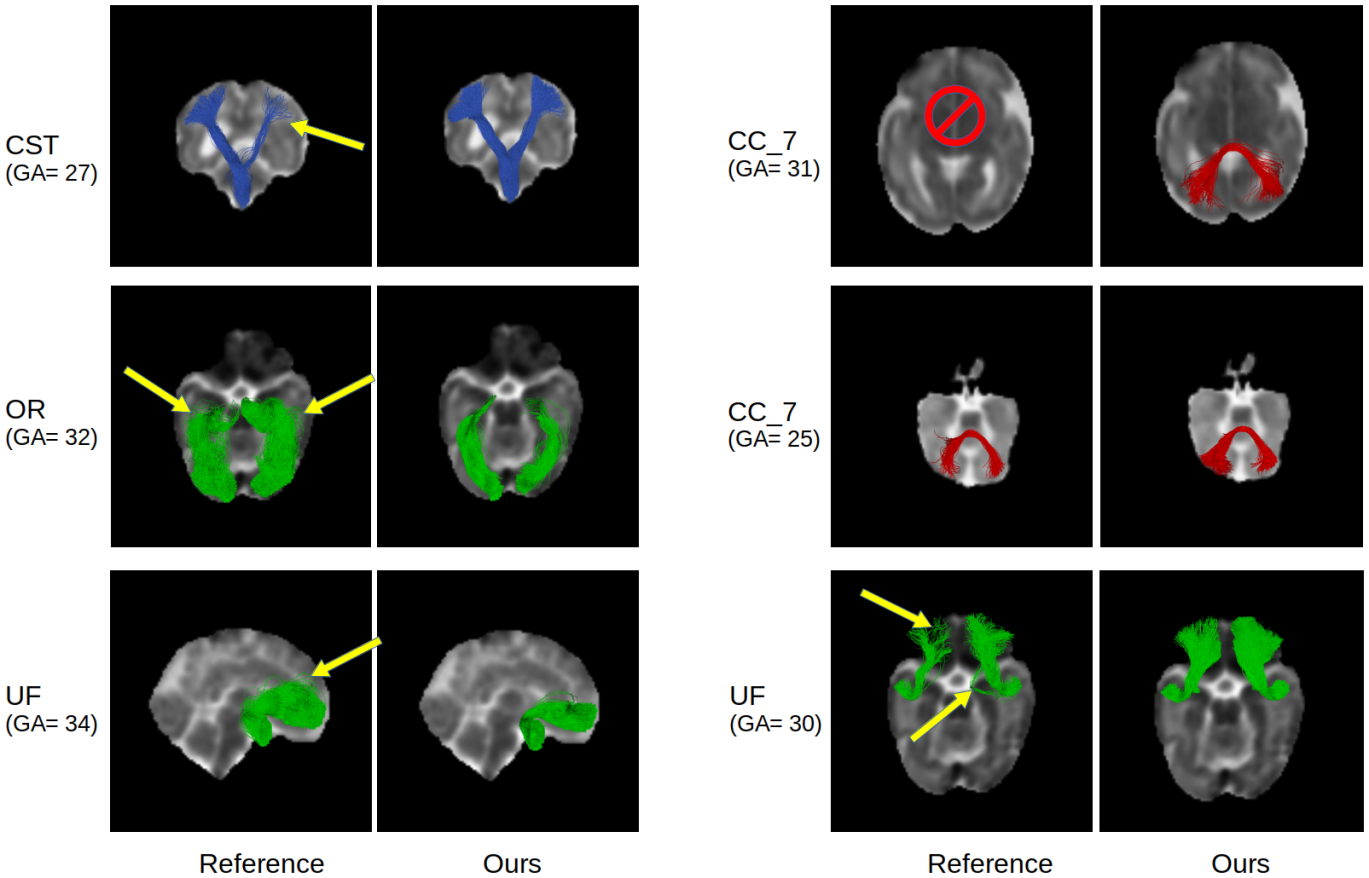
Example test results generated with our method and the reference method that was used to generate the streamlines on training images. Yellow arrows point to examples of incomplete tracts and spurious (false positive) streamlines. As shown in these examples, our method quite often performs better than the reference method and computes tracts that are more complete and have fewer false positives. In some cases, such as the top right example in this figure, the reference method completely failed to reconstruct the tract, while our method successfully reconstructed the same tract.

Overfitting and bias are persistent risks in all machine-learning methods. In the application considered in this work, overfitting and bias can arise in different ways. For example, the machine-learning method can memorize the overall shape of the tract and replicate the same tract shape on the test samples without considering the subject-specific input data. There is also the risk that the model may become biased to the specific characteristics of the training data, leading to poor generalizability on unseen or out-of-distribution test data. This is a common risk in medical imaging applications as image acquisition protocols can significantly influence the data distribution. To investigate the susceptibility of our method to these risks, we present additional results inFigures 13and14below.

**Fig. 13.**
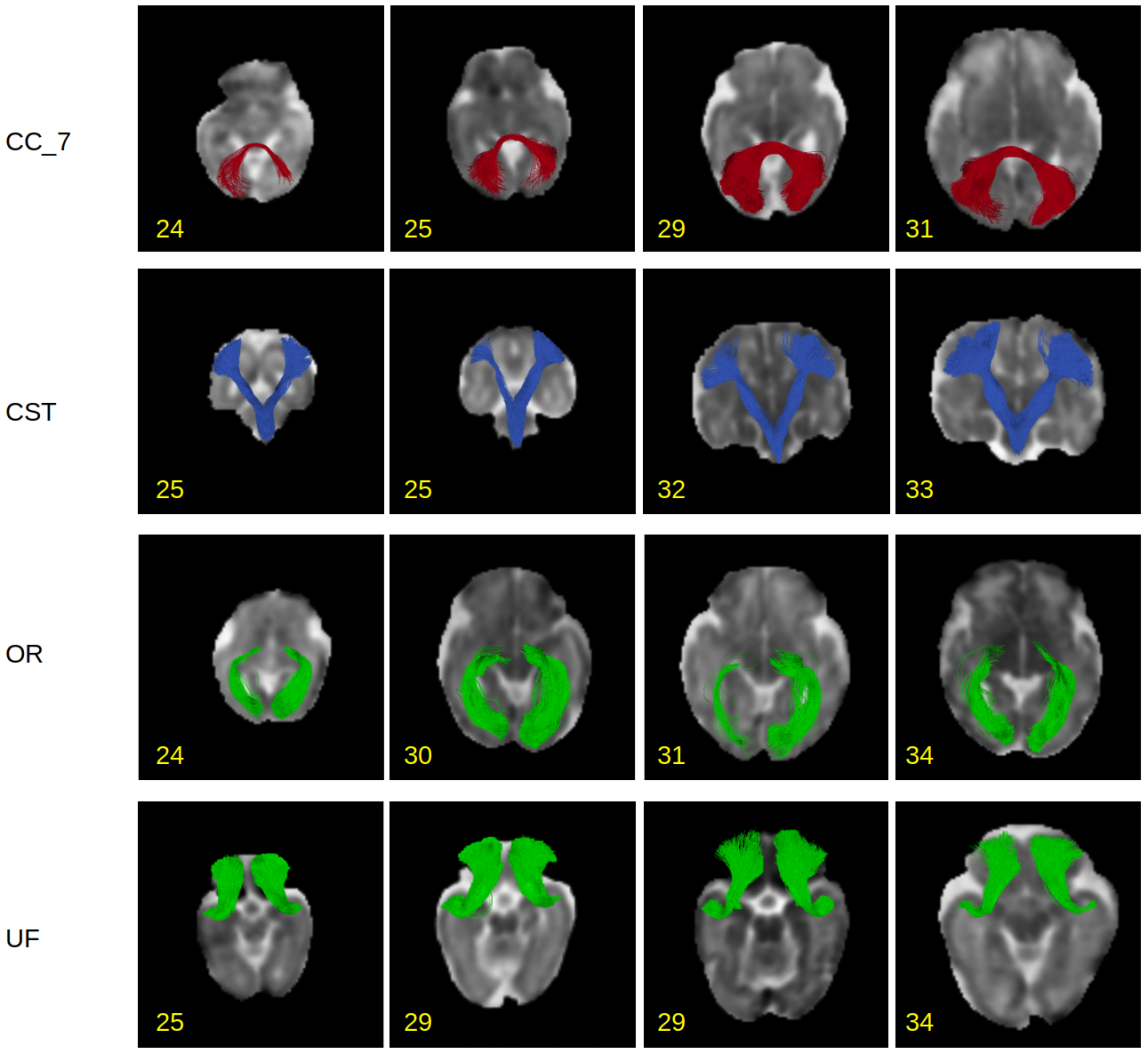
This figure shows the variability in the results computed by the proposed method. Each row in the figure shows a specific tract reconstructed by the proposed method for four different test subjects. These examples show that the proposed method can effectively learn, preserve, and reflect the inter-specific variability. The number at the lower left of each figure indicates the gestational age in weeks.

**Fig. 14.**
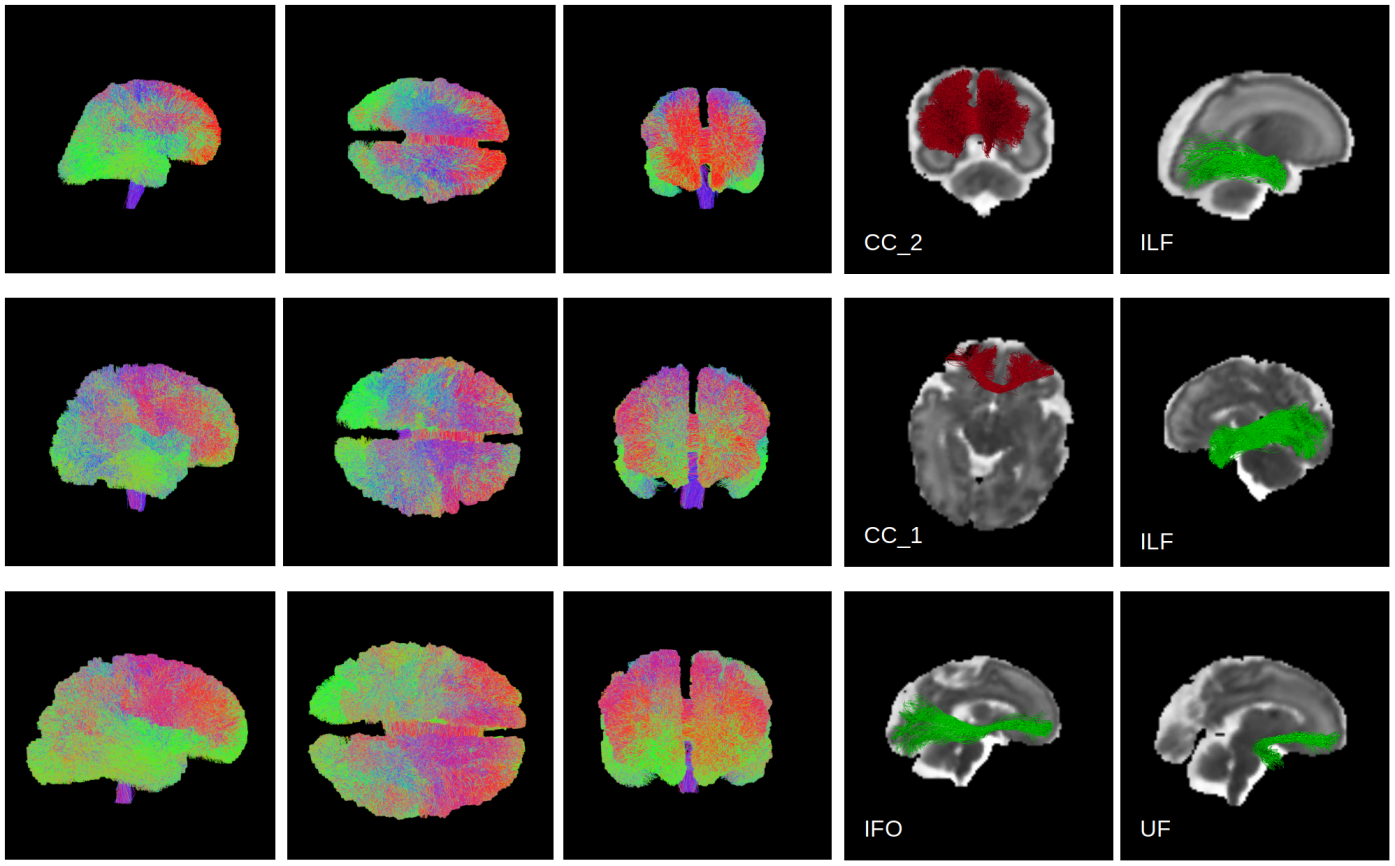
Tractography results computed by the proposed method on three example fetal subjects from the DHCP data. Each row presents a different subject. From left, the first three columns show the sagittal, axial, and coronal views of the whole-brain tractogram. The two right-most columns show example tracts extracted automatically with WMQL from each tractogram. The gestational ages of these fetuses from top to bottom are 27, 32, and 35 weeks.

Figure 13shows the results computed by our method for different test subjects. Specifically, it shows four different tracts (CC_7, CST, OR, and UF) reconstructed for four different test subjects. These results clearly show that our method computes results that are different for different test subjects. The shape, extent, and fullness of the tracts seem to be highly dependent on the subject data, which is an indication that our method does not memorize the training data. Instead, it learns to compute subject-specific streamlines on test samples such that the inter-subject variability is preserved and reflected in the results.

Figure 14shows results computed by our method on example fetal data from the Developing Human Connectome Project (DHCP). We applied our method on data from either of the two shells from the DHCP dataset and the results were almost indistinguishable, indicating that our method could generalize very well to data from different shells. The results presented inFigure 14have been computed with the data from the b = 1,000 s/mm^2^shell, which is quite different from our test data with b = 500 s/mm^2^. The results show that the proposed method can perform accurate tractography on out-of-distribution data. The whole-brain tractograms and individual tracts, extracted automatically using WMQL, look complete with very low false positives and false negatives.

### 3.3 Comparison with prior works

The significance of the advancements enabled by our method can be better appreciated by comparing our results with those of prior works. To this end,Table 2summarizes several prior studies that have attempted streamline tractography of the fetal brain. For each study listed in this table, we have briefly described the methodology followed to generate the tractography, the specific white matter tracts studied (if applicable), and a summary of the results. As this selection shows, past efforts have had limited success in reconstructing major white matter structures in the fetal brain. Interestingly, some of these studies have imaged post-mortem fetal brain specimens (ex-vivo) with specialized scanners. Many of them have also resorted to manual placement of ROIs to guide the tractography. Yet, most of these studies have achieved far inferior results compared with the results obtained by our method in this work. This comparison further points to the challenging nature of fetal tractography and to the significance of the results obtained with the new method.

**Table 2.** A summary of the methods and results for selected prior works on fetal brain tractography.

Ref.	METHODS	Tracts	RESULTS
Huang et al. (2009)	This study scanned postmortem fetal brains between 13 and 22 gestational weeks using 4.7T and 11.7T scanners. Tractography was performed using FACT with user-specified ROIs. The authors followed protocols designed for adult brains to specify ROIs for different tracts.	CC, UF, ILF, FX	The method reconstructed the tracts, but no success rate metrics were reported. Most tracts were partially reconstructed.
Kasprian et al. (2008)	The work computed tractography on fetal brains, scanned in utero, between 18 and 37 gestational weeks using 1.5 T scanners. Tractography was performed using FACT with manual specification of ROIs.	CC, CST, TC	Authors reported an overall success rate of 40%. Most reconstructed tracts showed only partial coverage of the anatomical extent.
Wilson et al. (2021)	Tracts were defined using multiple ROIs, including seed regions and inclusion/exclusion zones to confine tracts within one hemisphere. Tracts were refined by removing spurious streamlines via microstructure-informed filtering. Probabilistic streamline tractography was performed using multi-tissue spherical deconvolution. The experiments were conducted on 113 healthy fetuses with gestational ages ranging from 22 to 37 weeks, imaged with 3T scanners.	CST, OR, ILF, CC.	Tractography successfully generated most of the tracts, though OR was not identifiable at the earliest gestational stage. Often, the tracts demonstrated only partial anatomical coverage.
Song et al. (2015)	This study was performed on 10 postmortem fetal brains, imaged with 3T and 4.7T scanners, with subjects ranging in age from 15 to 22 gestational weeks. Tractography was performed using FACT. A two-ROI approach was employed to segment the streamline bundles.	FX, ILF, IFO, AF	Most reconstructed tracts showed only partial coverage of the anatomical extent of the tract.
Jakab et al. (2015)	A total of 20 normal and 20 abnormal fetal brains were scanned with 1.5 T scanners in utero between 22 and 36 gestational weeks. A white matter segmentation mask was used for seeding. A fourth-order Runge-Kutta method was used for tractography.	None	The study did not assess the tractography results in terms of individual tracts.
This study	A machine-learning model based on typical *in utero* fetal scans. The model is validated on an independent dataset of 11 fetal scans between 23 and 36 weeks of gestation.	ATR, CC _1_ , CC _2_ , CC _7_ , CST, IFO, ILF, OR, UF	All tracts are fully reconstructed across the gestational age. An expert rated all tracts with a mean score of “Good” or “Excellent”.

CC: corpus callosum; UF: uncinate fasciculus; ILF: inferior longitudinal fasciculus; FX: fornix; CST: corticospinal tract; TC: thalamocortical tracts.

The success of the new method developed in this work can be attributed to several factors. First, the proposed machine-learning model can synergistically combine different local and non-local information that are useful for tractography in a unified framework in the form of a deep neural network that can be optimized end-to-end. Even for fiber orientation, which is inherently a local information, our design effectively exploits the information in a large neighborhood around the current streamline tracing point. This reduces the impact of local fiber orientation estimation errors that can be significantly higher in fetal brain studies than in adult studies. Our method also effectively uses other sources of information that are difficult to incorporate into conventional tractography methods. It utilizes accurate tissue segmentation maps to launch the streamlines, guide the streamline propagation, and reject anatomically implausible streamlines. Another novel aspect of the proposed method was the incorporation of a streamline orientation prior in the form of a spatiotemporal fixel atlas that was precisely aligned to the subject brain. Lastly, we fed a long history of propagation directions, from up to 11 steps preceding the current point, as well as the distance to keypoints in the brain cortex to inform the tractography method of the location within the brain. This information was easily passed to the method via late fusion with other information at the final MLP model. The machine-learning model was trained on tractography results from 62 fetal brain scans across the gestational ages considered in this work. Therefore, unlike conventional methods that compute the next streamline propagation direction based purely on the local data from the given scan, our method computes the next step based on what it has learned after being trained on millions of streamlines from tens of subjects. Hence, our method represents an entirely different approach to streamline tractography, where local, non-local, and population-level information can be used jointly to address this challenging task.

### 3.4 Limitations and future directions

A limitation of the proposed tractography method is its reliance on the diffusion tensor model, which is incapable of resolving complex fiber configurations such as crossing fibers. There exist numerous alternative models that can resolve crossing fibers when sufficient dMRI measurements are available (Seunarine & Alexander, 2014). Application of these models on fetal data in prior works has been mostly limited to research-quality scans (e.g.,Wilson et al., 2021). In fetal imaging, due to the limited scan times and the overall low signal-to-noise ratio, the number and quality of measurements are too low to reliably estimate complex fiber configurations with advanced models. The diffusion tensor, despite its limitations, requires less data to estimate. Therefore, our use of the diffusion tensor model enhances the applicability of the proposed tractography method to cases where the number and quality of the diffusion measurements are limited. Note that our proposed method does not use the diffusion tensor at the current tractography point alone. Rather, we encode a large spatial context around the current tractography point, thereby giving the tractography method information about how the fiber orientations look like around the current point. This can help the tractography method, to some extent, determine the complex fiber configurations (i.e., crossing, fanning, bending) around the current point. Nonetheless, the use of the diffusion tensor model remains a limitation of our method that can be addressed in future works.

While we designed our method based on the requirements of fetal tractography, it may be useful for adult brain tractography as well. However, applying our method to adult brains would require a fixel atlas for adult brains, which was unavailable to us. On the other hand, recent years have witnessed a wide range of new tractography techniques, mostly targeting the adult brain, that may also benefit fetal brain tractography. These new techniques are predominantly based on machine learning (Karimi & Warfield, 2024;Poulin et al., 2019). Initial works used models such as multilayer perceptrons and recurrent neural networks to predict the next propagation step based on local dMRI data and immediate history of streamline propagation (Poulin et al., 2017;Wegmayr et al., 2018). More recent works have advocated for reinforcement learning approaches that, unlike supervised learning methods, do not depend on ground-truth streamlines for training (Sinzinger & Moreno, 2022;Théberge et al., 2021). Our preliminary experiments with reinforcement learning methods show that they may not be optimal for fetal tractography because they heavily rely on local fiber orientation information. Failure to exploit anatomical knowledge is a limitation of most tractography algorithms. It has been suggested that anatomical prior knowledge may be the key to improving all tractography methods (K. G. Schilling et al., 2020). Some of the recent machine learning-based tractography methods have demonstrated the important role of anatomical information (Cai, Lee, Newlin, Kerley, et al., 2023;Théberge et al., 2024). Future works can explore whether these techniques, such as reinforcement learning (Sinzinger & Moreno, 2022;Théberge et al., 2021,^2024^) or direct inclusion of structural MRI contrasts (Cai, Lee, Newlin, Kerley, et al., 2023), may improve fetal tractography as well.

Lastly, more comprehensive and more critical assessment of fetal brain tractography results is warranted. In this study, we used both objective quantitative metrics as well as subjective assessment by a human expert to achieve a balanced evaluation. Nonetheless, future studies may go further by assessing a larger number of white matter tracts and by evaluating the tractography of superficial white matter fibers such as the U-fibers. Future studies may also obtain evaluations from more than one human expert in order to account for inter-expert variability. Furthermore, important specific aspects of tractography results such as the gyral bias can be studied (Kleinnijenhuis et al., 2015;Reveley et al., 2015;K. Schilling et al., 2018). These evaluations can be enabled by comparison of dMRI-based tractography results with histological data.

## 4 Conclusion

This study presented a machine-learning approach to fetal brain tractography. Given the challenging nature of dMRI-based tractography in general, and the limited reliability of local fiber orientation computations in the fetal brain in particular, advanced machine-learning methods seem to be the natural solution to this problem. These methods are able to synergistically combine multiple sources of information to overcome the insufficiency of the local fiber orientation information. The method presented in this paper exploits the diffusion tensor image, propagation history, global spatial information, tissue segmentation, and a fixel atlas prior. We validated our model on an independent dataset consisting of 11 fetal subjects across gestational ages between 23 and 36 weeks, demonstrating that our method outperformed existing techniques in all evaluated tracts. A comparison with the prior art on fetal tractography shows that the results obtained with our method are substantially better than those reported in the literature. Improved tractography accuracy offered by our method can facilitate the tract-specific analysis of normal and abnormal fetal brain development with dMRI and the assessment of structural brain connectivity in utero.

## Data Availability

Sample data from this study may be shared at request. The code will be made available athttps://github.com/liuweide01/MLFT.
